# Elucidating brain transport pathways and cell type-dependent gene silencing of a durable lipid–siRNA conjugate administered into cerebrospinal fluid

**DOI:** 10.1093/nar/gkaf600

**Published:** 2025-07-02

**Authors:** Alexander G Sorets, Katrina R Schwensen, Nora Francini, Andrew Kjar, Adam M Abdulrahman, Alena Shostak, Ketaki A Katdare, Kathleen M Schoch, Rebecca P Cowell, Joshua C Park, Alexander P Ligocki, William T Ford, Lissa Ventura-Antunes, Ella N Hoogenboezem, Alex Prusky, Mark Castleberry, Danielle L Michell, Emma Fritsch, Sarah M Lyons, Timothy M Miller, Kasey C Vickers, Matthew S Schrag, Craig L Duvall, Ethan S Lippmann

**Affiliations:** Department of Biomedical Engineering, Vanderbilt University, Nashville, TN 37240, United States; Department of Chemical and Biomolecular Engineering, Vanderbilt University, Nashville, TN 37240, United States; Department of Biomedical Engineering, Vanderbilt University, Nashville, TN 37240, United States; Department of Biomedical Engineering, Vanderbilt University, Nashville, TN 37240, United States; Department of Biomedical Engineering, Vanderbilt University, Nashville, TN 37240, United States; Department of Neurology, Vanderbilt University Medical Center, Nashville, TN 37240, United States; Vanderbilt Brain Institute, Vanderbilt University, Nashville, TN 37240, United States; Department of Neurology, Washington University School of Medicine in St. Louis, St. Louis, MO 63110, United States; Department of Chemical and Biomolecular Engineering, Vanderbilt University, Nashville, TN 37240, United States; Department of Chemical and Biomolecular Engineering, Vanderbilt University, Nashville, TN 37240, United States; Department of Chemical and Biomolecular Engineering, Vanderbilt University, Nashville, TN 37240, United States; Department of Chemical and Biomolecular Engineering, Vanderbilt University, Nashville, TN 37240, United States; Department of Neurology, Vanderbilt University Medical Center, Nashville, TN 37240, United States; Department of Biomedical Engineering, Vanderbilt University, Nashville, TN 37240, United States; Department of Neurology, Vanderbilt University Medical Center, Nashville, TN 37240, United States; Department of Medicine, Vanderbilt University Medical Center, Nashville, TN 37240, United States; Department of Medicine, Vanderbilt University Medical Center, Nashville, TN 37240, United States; Vanderbilt Brain Institute, Vanderbilt University, Nashville, TN 37240, United States; Department of Chemical and Biomolecular Engineering, Vanderbilt University, Nashville, TN 37240, United States; Department of Neurology, Washington University School of Medicine in St. Louis, St. Louis, MO 63110, United States; Department of Medicine, Vanderbilt University Medical Center, Nashville, TN 37240, United States; Department of Neurology, Vanderbilt University Medical Center, Nashville, TN 37240, United States; Vanderbilt Brain Institute, Vanderbilt University, Nashville, TN 37240, United States; Department of Biomedical Engineering, Vanderbilt University, Nashville, TN 37240, United States; Department of Chemical and Biomolecular Engineering, Vanderbilt University, Nashville, TN 37240, United States; Department of Biomedical Engineering, Vanderbilt University, Nashville, TN 37240, United States; Department of Chemical and Biomolecular Engineering, Vanderbilt University, Nashville, TN 37240, United States; Department of Neurology, Vanderbilt University Medical Center, Nashville, TN 37240, United States; Vanderbilt Brain Institute, Vanderbilt University, Nashville, TN 37240, United States; Vanderbilt Memory and Alzheimer’s Center, Vanderbilt University Medical Center, Nashville, TN 37240, United States

## Abstract

The clinical neurosciences are in the midst of a renaissance spurred by the development of new therapeutic modalities. Short interfering RNAs (siRNAs), in particular, are gaining interest for treating neurological diseases owing to their capacity to sustain inhibition of nearly any gene target. However, to be effective, siRNA therapies must achieve delivery and on-target gene silencing activity in specific sites and cells in the brain. To this end, we developed a lipid–siRNA conjugate (L2-siRNA) that transports effectively throughout the brain when injected into cerebrospinal fluid (CSF). We provide a detailed examination of regional bulk tissue gene silencing in mice, highlighting potent knockdown 5 months after a single injection without detectable toxicity. Intrathecal delivery of L2-siRNA in rats further illustrates effective transport and knockdown using a clinically relevant route of administration. Single-cell RNA sequencing was additionally performed in mice to generate an atlas of cell type-specific knockdown. Lastly, we benchmarked L2-siRNA gene silencing activity in different brain regions against antisense oligonucleotides, a related but different gene silencing modality. Collectively, this work examines properties of lipid–siRNA conjugates that facilitate CSF to brain delivery and supports L2-siRNA as a promising platform for silencing genes implicated in central nervous system disorders.

## Graphical Abstract

**Graphical Abstract:**
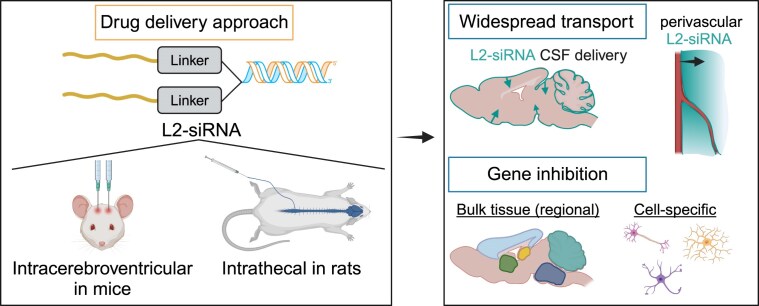

## Introduction

Advances in nucleic acid therapeutics have provided new avenues for treating long-intractable neurological diseases. The development of precise therapeutic modalities such as short interfering RNA (siRNA) has expanded the toolbox of medicines to correct disease-driving defects, as evidenced by the recent FDA approval of GalNAc conjugates that act in the liver [1]. In parallel, a deeper understanding of neurobiology has led to the identification of new gene targets for treating neurodegenerative diseases including but not limited to Huntington’s disease (HD), Alzheimer’s disease (AD), and spinocerebellar ataxias [2, 3]. As such, there is now a push to engineer siRNA technology to treat central nervous system (CNS) disorders, which requires effective delivery to brain regions and cell types where diseases manifest [4].

The most common injection route for nucleic acid therapies is into cerebrospinal fluid (CSF) because, at present, systemic delivery of siRNA-based therapeutics do not reach the CNS in meaningful concentrations due to the blood–brain barrier [5, 6]. Even with intrathecal drug delivery, however, the limited interface between bulk CSF circulation and brain parenchyma poses a considerable challenge for achieving therapeutic effects beyond the superficial layers of the brain adjacent to CSF. Transport to deeper brain structures is important because many diseases manifest in these sites, such as HD, which could prospectively be treated by lowering huntingtin (Htt) expression in medium spiny neurons of the striatum [7]. Yet, efficacious siRNA delivery to deep brain structures such as the striatum remains an unmet need for therapeutics administered into the CSF.

The central delivery challenge for siRNA drugs is co-optimizing cellular uptake with deep tissue penetration, two processes that are seemingly at odds with one another. For example, unconjugated siRNA structures administered carrier-free into CSF penetrate throughout the CNS but are not sufficiently internalized by cells to achieve potent gene silencing [8]. Conjugation of siRNA to lipophilic moieties is intended to improve cellular uptake through membrane interactions [9, 10]. However, cellular uptake can also counter transport; for example, conjugating cholesterol to an siRNA promotes robust cellular association, but generates steep concentration gradients around CSF–brain interfaces at the expense of delivery to deeper brains structures [11].

Given these challenges, the overarching objective of this study was to develop a lipid–siRNA conjugate with balanced cell uptake and tissue transport properties to achieve an ideal combination of brain tissue penetration, effective intracellular delivery, and durable, on-target gene silencing. We recently developed a lipid–siRNA conjugate termed “L2-siRNA” that was optimized in the context of navigating systemic delivery to tumors [12]. This prior work studied the effects of linker and lipid structure and revealed that a hydrophilic linker helped offset the lipid hydrophobicity that promoted potent cellular entry. The L2-siRNA conjugate exhibited balanced cell uptake and transport properties in serum, which was modulated by reversible shielding of the lipids due to albumin binding [12]. Here, we hypothesized that L2-siRNA could achieve effective transport through CSF compartments, dispersion through parenchymal tissue, and sufficient cellular uptake, while avoiding overt toxicity.

In this manuscript, we rigorously characterize and benchmark the L2-siRNA platform following administration into CSF, using intracerebroventricular (ICV) injection in mice and intrathecal injection in rats. Transport mechanisms were investigated, with an emphasis on deep brain structure delivery from subarachnoid (diffusion from brain surface) and perivascular (convective flow along vessels) CSF pathways [13–15]. Since neurodegenerative diseases manifest in specific regions and cell types, we profiled spatial variation in brain and spinal cord delivery as well as target gene silencing potency and longevity. Single-cell RNA sequencing (scRNA-seq) was further applied to examine cell-type dependent gene silencing activity. Building on the emerging role of CSF-interfacing brain border cells in health and disease, we completed a unique and deep exploration of the ability of L2-siRNA to silence genes in leptomeningeal fibroblasts, choroid plexus epithelial cells, and border-associated macrophages. Last, we provide a head-to-head comparison of L2-siRNA and an antisense oligonucleotide (ASO), a more clinically mature modality than siRNAs with a track record for treating CNS and other diseases. Collectively, this work establishes L2-siRNA as a promising platform for gene silencing across cell types and regions of the CNS.

## Materials and methods

### Synthesis of oligonucleotides

Oligonucleotide syntheses were performed using standard solid-phase chemistry with a MerMade 12 automated RNA synthesizer (BioAutomation) on controlled pore glass with a universal support (1 or 10 μmol scale, 1000 Å pore), using 2′-F and 2′-OMe phosphoramidites with standard protecting groups (Glen Research). 5′-(*E*)-Vinylphosphonate (VP) was incorporated on the 5′ terminus of the antisense strand using POM-vinyl phosphonate 2′-OMe-uridine CE-phosphoroamidite (LGC Genomics). All strands were grown on the universal support except for Cy5-labeled oligos, which were synthesized on Cy5-functionalized 1000 Å CPG (Glen Research). Amidites were all dissolved at 0.1 M in anhydrous acetonitrile, except for 2′-OMe uridine, which requires 20% dimethylformamide as a co-solvent. All nucleic acid sequences are listed in Supplementary Table S1. The ASO^Htt^ sequence was designed elsewhere [16] and ordered from IDT.

### Cleavage and deprotection of oligonucleotides

Unconjugated and conjugated sense strands were cleaved and deprotected with a 1:1 solution of 28%–30% ammonium hydroxide and 40% aqueous methylamine (AMA) for 2 h at room temperature. Cy5-labeled oligonucleotides were cleaved in 28%–30% ammonium hydroxide at room temperature for 20 h. The VP-containing antisense strands were cleaved and deprotected by treating the CPG with a 3% diethylamine (DEA) solution in 28%–30% ammonium hydroxide (20 h, 35°C).

### Purification and characterization of oligonucleotides

After cleavage and deprotection, oligonucleotides were dried under vacuum to remove solvents (Savant SpeedVac SPD 120 Vacuum Concentrator, Thermo Fisher). Pellets were resuspended in water and purified on a Waters 1525 EF HPLC system equipped with a Clarity Oligo-RP column (Phenomenex) under a linear gradient [60% mobile phase A (50 mM triethylammonium acetate in water) to 90% mobile phase B (methanol)]. Cy5-labeled and unconjugated sense strands were first desalted using Gel-Pak column (Glen Research), followed by chromatography under a linear gradient (85% to 40% mobile phase A). Oligonucleotide fractions were dried, resuspended in nuclease-free water, sterile filtered, and lyophilized. Dimethoxytrityl (DMT) protecting group was removed from the purified, dried, unconjugated strand using 20% acetic acid for 1 h at room temperature, followed by desalting.

Antisense strands were purified over a 10 mm × 150 mm Source 15Q anion-exchange column (Cytiva) using a gradient of sodium perchlorate. Buffer A consisted of 10 mM sodium acetate in 20% acetonitrile and buffer B consisted of 1 M sodium perchlorate in 20% acetonitrile. The run conditions were 90% to 70% buffer A over 30 min at a flow rate of 5 ml/min. Purified oligonucleotides were desalted, sterile filtered, and lyophilized.

The identity of oligonucleotides was verified by liquid chromatography–mass spectrometry (LC–MS, Thermo Fisher LTQ Orbitrap XL Linear Ion Trap Mass Spectrometer). LC–MS was performed using a Waters XBridge Oligonucleotide BEH C18 Column under a linear gradient [85% phase A (16.3 mM triethylamine–400 mM hexafluoroisopropanol) to 90% phase B (methanol)] run for 10 min at 45°C.

### *In vitro* assessment of carrier-free oligonucleotide uptake

Uptake propensity of siRNA conjugates was assessed by treating N2a neuroblastoma cells with Cy5-labeled compounds and measuring fluorescence by flow cytometry. In brief, N2a cells (<P21) were seeded at 100 000 cells/ml onto uncoated 24-well plates and allowed to adhere overnight. The cells were then treated with siRNA-Chol, L2-siRNA, or free siRNA in serum-free Opti-MEM at 60 nM (Cy5 concentration) for 2 h. Unbound compound was removed with two Dulbecco's Phosphate-Buffered Saline -/- (DPBS) washes and then the cells were dissociated using Accutase (Sigma). The collected cells were pelleted and resuspended in flow cytometry buffer [0.5% bovine serum albumin (BSA) in DPBS without calcium and magnesium] to run on a Guava EasyCyte (Luminex) flow cytometer. The analysis was performed in FlowJo™ v10.8 Software (BD Life Sciences) to gate single cells (from debris and doublets) and measure geometric mean fluorescence intensity from over 2000 cellular events.

### *In vitro* assessment of carrier-free gene silencing

*In vitro* gene silencing of oligonucleotide conjugates was assessed by carrier-free reverse transfection. Wells were prepared with 250 nM of siRNA in Opti-MEM, and then 75 000 N2a cells were added to each well (24-well plate). After 24 h, an equal volume (500 μl) of full serum Dulbecco’s modified Eagle’s medium (10% fetal bovine serum) was added to each well and after an additional 24 h, cells were harvested for reverse transcription quantitative polymerase chain reaction (RT-qPCR).

### Animal husbandry

Adult C57BL/6J male mice were ordered from Jackson Laboratory and used between 10 and 16 weeks of age. Mice were housed continuously in an environmentally controlled facility in a 12-h light/dark cycle with *ad libitum* access to food and water. All mouse protocols were approved by the Institutional Animal Care and Use Committee (IACUC) at Vanderbilt University. One-month rat intrathecal studies were performed in male Sprague Dawley rats at Vanderbilt and approved by Vanderbilt's IACUC. All 48-h rat studies were conducted in 69–84-day-old Sprague Dawley rats (male and female) and approved by the IACUC at Washington University in St. Louis.

### ICV injections and euthanasia

siRNA duplexes were annealed in 0.9% sterile saline by heating to 95°C and gradually cooling to 4°C on a thermocycler. On the day of injection, the compounds were concentrated using a 3K Amicon Ultra spin filter (UFC500324). The concentrations were measured by absorbance (260 nm) on a NanoQuant Tecan plate reader and adjusted as necessary by adding saline.

Mice were anesthetized with isoflurane and mounted on the stereotactic rig, where they received continuous isoflurane for the duration of the surgery. Eye ointment (Bausch^+^ Lomb) was administered to prevent drying and then the scalp was sanitized with betadine and 70% ethanol, alternating three times each. The scalp was opened with a midline incision and hydrogen peroxide was applied to expose bregma. Injection coordinates, as distance from bregma, were ±1 medial–lateral, −2.3 dorsal–ventral, and −0.2 anterior–posterior. Two holes were drilled through the skull at these coordinates for bilateral injection. The syringe (Hamilton Model 701, blunt 30 G) was brought to these coordinates and slowly lowered into the ventricle. Injections were performed at 1 μl/min for a total of 5 μl per ventricle. To minimize backflow, the needle was left in the ventricle for an additional 5 min prior to gradual retraction. The scalp was then sutured shut, and mice were monitored for their recovery. To maintain body temperature, mice were placed on a heating pad (37°C) during and after surgery. For analgesia, mice received an intraperitoneal injection of ketoprofen (5–10 mg/kg Ketofen, Zoetis) prior to surgery and daily for 72 h post-operation.

At the terminal time point, mice were euthanized by ketamine (450 mg/kg)/xylazine (50 mg/kg) overdose and transcardially perfused with cold heparinized (10 U/ml) DPBS−/− to remove blood cells from the vasculature. For flow cytometry studies, the right hemisphere was extracted into DPBS and processed into single cells as described below. For gene silencing studies, brains were extracted after perfusion and cut into 1-mm slices using a sagittal brain matrix. Biopsy punches (2 mm) were taken from different brain regions (hippocampus, striatum, cerebellum, posterior cortex, brainstem) and stored in RNAlater (Thermo AM7020) for downstream analyses. Spinal cords were extracted by extrusion with Hanks' Balanced Salt Solution (HBSS) and segmented into cervical, thoracic, and lumbar regions using the characteristic enlargements as a guide. For biodistribution studies, mice were further perfused with 4% paraformaldehyde (PFA), and then brains and spinal cords were extracted, immersion fixed in 4% PFA overnight at 4°C, and subjected to further downstream histological processing. Organs were also harvested and Cy5 fluorescence was measured by IVIS Lumina III imaging (Caliper Life Science, Hopkinton, MA).

### Rat intrathecal injections and euthanasia

Rat intrathecal administration of siRNA conjugates was performed as described [17] by inserting a catheter into the cauda equina space and injecting a bolus of L2-siRNA. To prepare for surgery, rats were anesthetized with isoflurane, shaved, and disinfected by alternating betadine and 70% ethanol for three cycles. After locating the injection site between L6 and L5, an incision was made in the skin and connective tissue was removed by blunt dissection. Local anesthetic (50 μl of 0.5% bupivacaine) was applied intramuscularly near the spinal column and allowed to settle before inserting the guide cannula (19G blunted needle) into the spinal column. Next, a catheter was threaded through the guide cannula and into the cauda equina space. Successful placement of the catheter was confirmed by visualization of CSF backflow. A bolus dose of L2-siRNA (30 μl, ∼1 μl/s) was administered, followed by a 40 μl saline flush. The catheter was then heat-sealed and the incision was closed with staples or sutures. Rats then received carprofen (5 mg/kg intraperitoneal) and were monitored for recovery on a warming pad. For 48-h biodistribution studies, rats were euthanized with Fatal Plus (200 mg/kg) and perfused with heparinized PBS (10 U/ml). For 1-month studies, rats were perfused with DPBS (−/−) under isoflurane.

### Peptide nucleic acid hybridization assay

We adapted a previously described peptide nucleic acid (PNA) assay to quantify absolute siRNA delivery after ICV administration in mice and intrathecal administration in rats [18, 19]. To prepare homogenates, tissue biopsy punches were removed from RNAlater, placed in 300 μl homogenization buffer (Thermo, QS0518) plus proteinase K (Thermo, QS0511, 1:100), and disrupted using a Tissuelyzer 2.0 for 5 min at 30 Hz. Following a 1-h incubation at 65°C, the samples were spun down at 15 000 × *g* for 15 min and the supernatant was collected for storage at −80°C. The standard curve was prepared at the same time as the homogenates, with a maximum of 10 000 fmol and minimum of 156.25 fmol by 1:2 serial dilution. A new standard curve was prepared for each conjugate type and siRNA sequence.

Samples were thawed and sodium dodecyl sulfate (SDS) (component of homogenization buffer) was precipitated from 200 μl of homogenate with 20 μl of 3 M potassium chloride and centrifuged at 4000 × *g* for 15 min. The supernatant was collected and centrifuged at the same speed for an additional 5 min to ensure complete removal of the precipitate. If samples required a dilution to fall within the standard curve, they were diluted to 200 μl in homogenization buffer prior to precipitation. Next, 150 μl of supernatant was transferred to a screw cap tube, where 100 μl of hybridization buffer (50  mM Tris, 10% ACN, pH 8.8) and 2 μl of 5 μM PNA probe (∼10 pmol/150 μl of sample, PNA bio) were added. The probe was annealed to the antisense strand by heating to 90°C and then 50°C for 15 min each. The samples were then run through a DNAPac PA100 anion-exchange column (Thermo Fisher Scientific) on an iSeries LC equipped with RF-20A fluorescence detector (Shimadzu). Mobile phases consisted of buffer A (50% acetonitrile and 50% 25 mM Tris–HCl, pH 8.5; 1 mM ethylenediaminetetraacetate in water) and buffer B (800 mM sodium perchlorate in buffer A), and a gradient was obtained as follows: 10% buffer B within 4 min, 50% buffer B for 1 min, and 50%–100% buffer B within 5 min [20]. The final mass of siRNA was calculated using the area under the curve of Cy3 fluorescence from a standard curve of known quantities of siRNA or L2-siRNA spiked into untreated tissue homogenates.

### RT-qPCR

RT-qPCR methodology was employed to determine mRNA silencing *in vitro* and *in vivo*. For *in vitro* studies, cells were lysed in RLT buffer plus β-mercaptoethanol (1:100) and RNA was extracted using an RNeasy plus mini kit (Qiagen, 74134). Reverse transcription into complementary DNA (cDNA) was performed according to iScript manufacturer instructions (iScript cDNA synthesis kit, Bio-Rad, 1708891). Gene expression was measured by TaqMan qPCR on the cDNA using 20 μl reactions, run on a Bio-Rad CFX96, and analyzed in CFX Maestro software.

For *in vivo* studies, brain homogenates were prepared in 350 μl of RLT buffer plus β-mercaptoethanol and processed with 5-mm stainless steel beads (Qiagen, cat. no. 69989) for 5 min at 30 Hz (TissueLyser II). RNA was then extracted using an RNeasy plus micro kit (Qiagen, 74034) according to manufacturer’s instructions. RNA was eluted in 14 μl RNAse-free water and the concentration and purity were measured by 230/260/280 nm absorbance on a NanoDrop 2000c spectrophotometer. Last, qPCR was performed by preparing a 10 μl reaction mixture composed of 2× master mix, water, TaqMan probes, and cDNA sample. TaqMan probes were Mm00478295_m1 (*Ppib*), Mm01213820_m1 (*Htt*), Rn00577462_m1 (Htt), Rn03302274_m1 (*Ppib*), and Mm02619580_g1 (*Actb*). *In vivo* samples were run on a Quant 12k flex in triplicate (2 min at 50°C, 10 min at 95°C, and then cycle 15 s at 95°C and 1 min at 60°C). All samples were analyzed according to standard ΔΔCt methodology. Each sample is normalized to *Ppib* as a housekeeping gene unless *Ppib* is the target of the siRNA, in which case *Actb* is the housekeeping gene. Conventional RT-qPCR controls (no-template control and no reverse transcriptase control) were run on every plate and did not amplify.

### Western blotting

Brain homogenates were prepared in freshly made lysis buffer containing 10 mM HEPES (Sigma, adjusted to pH 7.2), 250 mM sucrose (Sigma), 1 mM ethylenediaminetetraacetic acid (EDTA), 1 mM sodium fluoride, 1 mM sodium orthovanadate, and 1 protease inhibitor tablet (Roche, 11836170001). Seventy-five microliters of buffer was added to each biopsy punch, and samples were homogenized three times for 10 s with a handheld homogenizer. Samples were cooled on ice for 30 s between homogenizations to prevent protein degradation. All homogenates were then sonicated (handheld) for 20 s to liberate nuclear proteins, and samples stored at −80°C until further analysis. Bicinchoninic acid (BCA) assay (Thermo, 23225) was used to quantify protein and normalize loading of each sample. In brief, 10 μl of sample was added to each well in duplicate, followed by the addition of 200 μl of substrate. The plate was incubated for 30 min at 37°C in the dark. Absorbance was measured at 562 nm and protein was quantified from a bovine serum albumin standard curve.

To prepare samples for western blotting, 10 μg of each sample was diluted with Tris-buffered saline (TBS) in XT sample buffer (Bio-Rad, 1610791) plus 5 mM dithiothreitol (DTT) and β-mercaptoethanol (1:10) and heated for 5 min at 95°C to fully denature proteins. Samples were loaded on a 3%–8% Tris-acetate gel (Bio-Rad, 3450131) in Tricine running buffer (Bio-Rad, 1610790) or Novex tricine SDS running buffer (LC1675). The gel was run at 120–150 V until the loading dye ran off the bottom. Next, the gel was transferred to a nitrocellulose Midi membrane (Bio-Rad, 1704159) using a Trans-Blot Turbo (Bio-Rad). This membrane was then blocked for 1 h in 5% non-fat milk (Bob’s Red Mill or Fisher 50-488-785) diluted in TBS. Primary antibodies were added overnight in 5% milk in TBS + 0.1% Tween 20 (TBST): huntingtin (Clone D7F7, Cell Signaling, 5656, 1:1000, for mouse blots), huntingtin (Abcam, 1:5000, ab109115, for rat blots), beta-actin (Clone, 13E5, Cell Signaling, 4970, 1:2000), and GFAP (Clone, D1F4Q, Cell Signaling, 12389, 1:2000). After rocking at 4°C overnight, the membrane was washed four times for 10 min with TBST. The HRP-conjugated secondary antibody (Abcam, ab6721) was diluted 1:20 000 in TBST and applied at room temperature for 1 h. When applicable, GAPDH (HRP-60004, Proteintech, 1:3000) was added with the secondary. After washing thrice with TBST and once with TBS for 10 min, the HRP substrate was added to visualize the bands. For huntingtin detection, SuperSignal West Femto Substrate (Thermo, 34095) was used, while Clarity Western ESC (Bio-Rad, 1705060) was used for beta actin detection. Blots were imaged for chemiluminescence on a Bio-Rad ChemiDoc MP imaging system and analyzed in ImageLab to measure total band intensity.

### Histology

To prepare fixed brains for frozen sections, sucrose gradients were performed for cryoprotection by immersion in 15% sucrose for 24 h (or until the sample sinks), followed by immersion in 30% sucrose for an additional day. The hemispheres were then embedded in Epredia Neg-50 medium, sectioned to 30 μm on a cryostat, and stored at −80°C until staining.

Antibody staining was performed as follows. First, samples were thawed to room temperature and a barrier was drawn using a hydrophobic pen to localize the staining reagents on the sample. Samples were washed in DPBS−/− with 0.3% Triton X-100 (PBST), followed by blocking for 1 h at room temperature in PBST plus 5% donkey serum (Sigma, D9663). Slides were then incubated either 2 h at room temperature or overnight at 4°C with the primary antibody diluted in DPBS containing 1% BSA and 0.5% Triton X-100. Primary antibodies include CD31 (1:100, BD Biosciences, 550539), Lyve1 (1:200, R&D, AF2125), AQP4-488 (1:500 for overnight or 1:200 for 2 h, Abcam, ab284135), Glut1-PE (1:300, Abcam, ab209449), and AQP1 (1:200, Abcam, ab168387). The sections were then washed three times in PBST for 5 min, followed by a 1-h room temperature incubation in the appropriate secondary antibody (1:500 dilution in DPBS containing 1% BSA and 0.5% Triton X-100). Sections were washed, then incubated with DAPI (1:5000–1:10 000, 5 mg/ml stock, Thermo, D1306) for 10 min, washed again in DPBS, and then mounted under a coverslip with ProLong Gold antifade reagent. Imaging was performed either on a Leica epifluorescence microscope (tiled images of entire brain) or a Zeiss LSM 710 confocal microscope (higher magnification of select regions). Image processing was performed using Fiji software. For samples where antibody staining was not required (i.e. assessing Cy5-tagged siRNA signal), slides were washed twice with DPBS and then mounted with ProLong Gold Plus DAPI.

Toxicity immunohistochemistry was performed on paraffin-embedded sections using the Epredia Autostainer 360. Sections were baked at 60°C for 1 h, and then deparaffinized and rehydrated through xylene and ethanol steps. Antigen retrieval was performed using the Epredia PT module in citrate buffer (pH 6.0) at 97°C. Slides were then transferred to the autostainer and blocked with Dako Protein Block (X0909) and Flourescent Block (Thermo Fisher, 37565). Primary antibodies were diluted in Dako protein block and incubated on the slides for 1 h at room temperature. Astrocytes were labeled with anti-GFAP (1:1000, Dako, Z0334) and microglia with anti-Iba1 (1:500, Wako, 019-19741). Slides were then washed and incubated in Alexa Flour 647-conjugated secondary antibody (1:1000, Thermo Fisher, A21245) for 1 h at room temperature. Sections were mounted with ProLong Gold with DAPI (Invitrogen, P36931) and imaged using the Aperio Versa 200 slide scanner. All quantification was done using ImageJ.

### Tissue clearing

Mouse brains or spinal cords were incubated for 3–5 days in 4% PFA and the right hemispheres were embedded in CLARITY polyacrylamide hydrogel (4% acrylamide, 0.05% bis-acrylamide, 0.25% temperature-triggering initiator VA-044 in 0.1 M PBS). To allow time for the polyacrylamide to permeate, the tissue remained in unpolymerized solution at 4°C for 2 weeks. Tubes were then moved to a 37°C water bath for 4 h to activate the VA-044 acrylamide crosslinker and polymerize the hydrogel. After the polymerization, tissue was cleared passively with a clearing solution [200 mM boric acid, 4% (w/v) SDS, pH 8.5] at 37°C shaking in a humidified incubator for 4–8 weeks. To stain for vasculature, the sample was washed overnight in PBS (0.1 M) with 0.1% Triton X-100 and then incubated in the same buffer containing lectin-fluorescein (0.5 mg/ml, FL-1171, Vector Laboratories), overnight for spinal cords and 2 days for brains. The same wash was repeated overnight, and afterward the samples were incubated in 68% thiodiethanol (TDE) overnight for refractive index matching (1.33). The samples were imaged on a light-sheet Z1 microscope (Zeiss) using 20× objective illumination and processed with Imaris software (version 9.9.0, Bitplane, USA).

### Fast protein liquid chromatography

Fast protein liquid chromatography (FPLC) was used to assess binding of different compounds to albumin in human CSF. Cy5-labeled conjugates or free siRNA (1 μM) was mixed with 300 μl post-mortem human CSF or human serum albumin (7.5 μM). After 30 min of incubation at 37°C, the volume was then brought to 1 ml in running buffer (10 mM Tris–HCl, 0.15 M NaCl, 0.2% NaN_3_), filtered (0.22 μm, Millipore, UFC30GV00), and fractionated by size exclusion through three tandem Superdex 200 Increase columns on an Akta Pure FPLC (GE Healthcare). The siRNA content of each fraction was measured with a plate reader (Biotek Synergy H1) for Cy5 fluorescence.

### Flow cytometry

Harvested brain tissue was converted to single-cell suspensions using the papain-based mouse adult brain dissociation kit from Miltenyi Biotec (130-107-677), with appropriate steps for myelin removal and red blood cell lysis according to the manufacturer’s instructions. Cells were then FcR-blocked for 10 min on ice (10 μl per sample, Miltenyi, 130-092-575). The cells from each brain were then split into four experimental samples—one for each cell-specific antibody cocktail: Thy1 for neurons (1:5000; Biotechne, FAB7335P), ACSA2 for astrocytes (1:2000; Miltenyi, 130-123-284), CD11b for immune cells (1:2000; BD Biosciences 561689), and CD31 for endothelial cells (1:2000; eBioscience, 17-0311-80). Each population was additionally stained with O1 (1:100, R&D Systems, FAB1327G), a marker for oligodendrocytes, which are excluded from analysis. After washing, cells were resuspended in DPBS−/− with 0.5% BSA and DAPI (1:10 000, 5 mg/ml stock) and run on an Amnis CellStream flow cytometer. Twenty-five thousand cellular events were recorded. The gating scheme to identify Cy5-positive cells leveraged fluorescence-minus-one (FMO) controls, which are samples stained with identical antibodies to the experimental groups but lack Cy5 signal (originating from an uninjected mouse). Single color compensation controls were run for each fluorophore. To validate ACSA2 gating of astrocytes, cells were sorted on a four-laser BD FACSAria III and RT-qPCR was performed as described using TaqMan probes to show enrichment of astrocytes (Mm01253033_m1) and depletion of endothelial cells (Mm00727012_s1).

### Multiplexed analysis of cytokines

Mice were administered 15 or 5 nmol of L2-siRNA by ICV injection as previously described. Two weeks after ICV injection, the cortex and striatum were biopsy punched, flash frozen, and stored at −80°C. To prepare homogenates, samples were thawed in cell lysis buffer (Bio-Rad, 171304006M) and homogenized three times for 10 s with a handheld homogenizer, with 30-s intervals on ice between homogenizations. The solution was then sonicated for 30 s and centrifuged at max speed for 10 min at 4°C. Supernatant was collected and total protein was quantified by BCA assay as previously described. The samples were then diluted to 1 mg/ml and processed by Eve Technologies using the Mouse High Sensitive 18-Plex Discovery Assay according to the manufacturer’s protocol (Millipore Sigma).

### scRNA-seq sample preparation

Mice were administered 15 nmol of L2-siRNA by ICV injection as previously described. The siRNA sequence was targeted to *Ppib* or a nontargeting control. After 1 month, mice were perfused with heparinized (10 U/ml) DPBS and a single-cell suspension of the brain was prepared using the adult brain dissociation kit as previously described. Cd11b-positive and -negative cells were isolated via MACS sorting with Cd11b-coated microbeads (Miltenyi, 130-097-142) according to the manufacturer’s instructions. After two washes with cell suspension buffer, cells were counted on a hemocytometer and diluted to target 2000 (Cd11b^pos^) or 20 000 (Cd11b^neg^) cells for encapsulation. Unencapsulated cells were used for RT-qPCR as previously described. The single-cell libraries were prepared using the PIPseq T2 v3.0 (for Cd11b^pos^) or v3.0 PIPseq T20 kits (for Cd11b^neg^) following manufacturer’s specifications. Reads were generated at a depth of 40 million reads for CD11b^pos^ and 400 million reads for CD11b^neg^ from an Illumina NovaSeq 6000 PE150 sequencing run, yielding a final read depth of 20K reads per cell.

### scRNA-seq data processing

Reads were processed using the PIPseeker alignment algorithm (v02.01.04). Count matrices were then processed using standard techniques in Seurat (v4). Datasets were merged without batch correction, and cells were filtered based on number of features, keeping cells with 800–4000 uniquely expressed genes for samples processed with T2 kits and 800–10 000 for samples processed with T20 kits. Counts were then normalized, and the dataset was reduced to the top 2000 variable genes. The data were then scaled, and dimension reduction was performed with Principle Component Analysis (PCA) and Uniform Manifold Approximation and Projection (UMAP) based on the first 50 principal components to visualize the data. Cells were clustered using the Louvain algorithm and annotated based on standard marker genes according to the mouse brain atlas [21]. Average *Ppib* expression levels were calculated using the AverageExpression() function in Seurat and were reported per cluster per biological replicate. For barplots, expression levels were normalized to L2-siRNA^NTC^ control.

### Statistical analysis

ANOVA was used for comparisons involving more than two groups with the conservative Bonferroni’s approach to correct for multiple comparisons. Unpaired two-tailed *t*-tests were used for all comparisons involving two groups. When assessing knockdown, different brain regions were considered independent tests.

## Results

### Lipid–siRNA conjugate structure and properties

**Figure 1.**
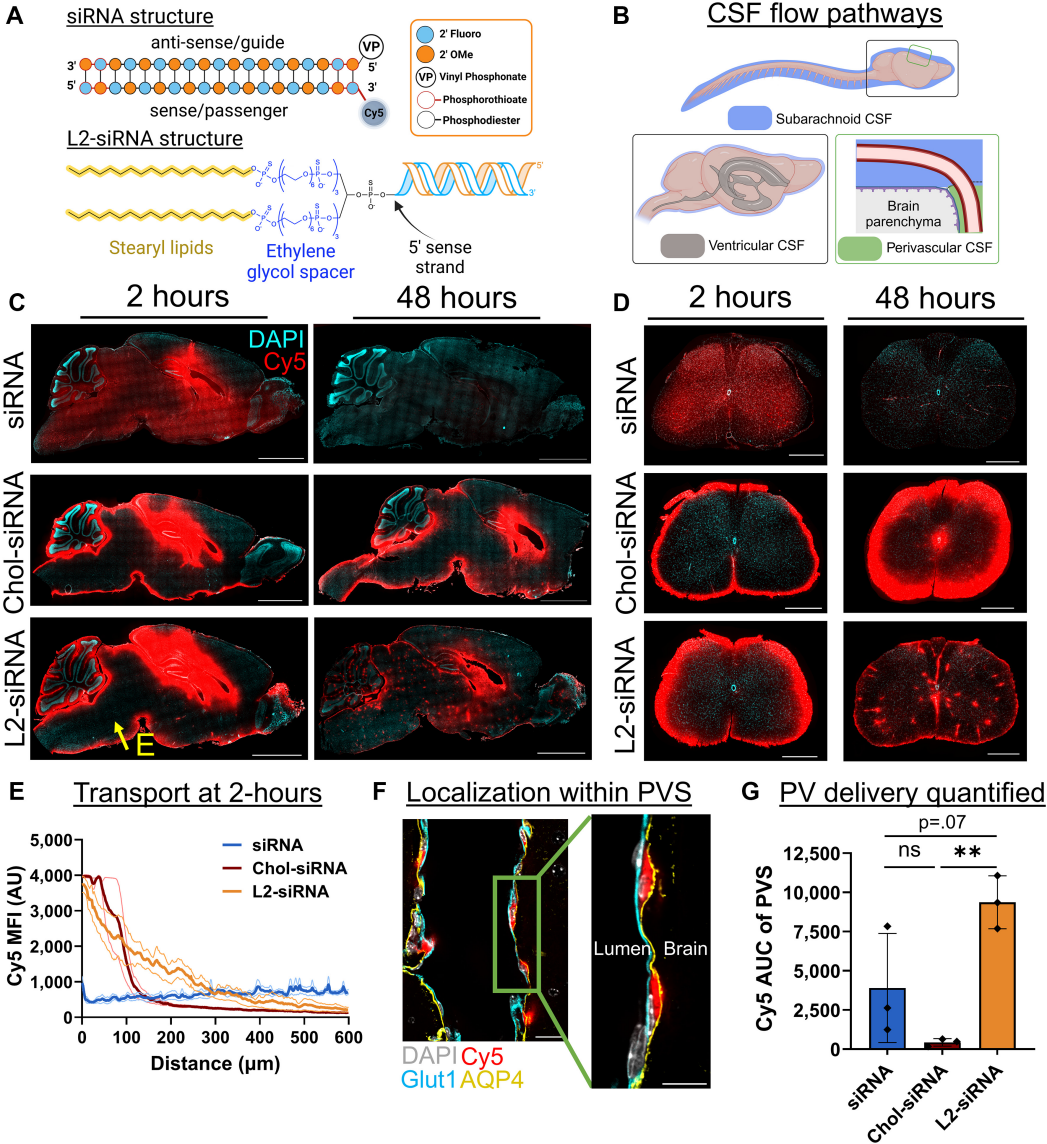
Lipid–siRNA conjugate structure determines CSF to brain transport. (**A**) All nucleic acid structures are synthesized with alternating 2′-*O*-methyl (OMe) and fluoro bases, terminating with two phosphorothioate bonds. All sequences utilize the 5′ vinyl phosphonate (VP) on the antisense strand, and when applicable the Cy5 fluorophore is positioned at the 3′ position of the sense strand. Lipid conjugates are attached to the 5′ end of the sense strand. L2-siRNA is composed of two 18-carbon lipid tails each connected to the siRNA through an 18-ethylene glycol unit spacer. Chol-siRNA (structure not shown) contains a triethylene glycol spacer separating the cholesterol moiety from the siRNA. (**B**) Overview of CSF anatomy. To examine biodistribution, Cy5-tagged compounds are injected into CSF through the ICV route. CSF flows from the ventricular system to the subarachnoid space, and then some CSF moves alongside vessels in the perivascular spaces of the brain. (**C**) Histological examination of CNS distribution either 2 or 48 h after ICV injection of unconjugated siRNA, Chol-siRNA, or L2-siRNA (7.5–10 nmol dose). Representative sagittal images of left hemispheres are displayed (*N* = 3 mice per condition). Additional biological replicates are shown in Supplementary Fig. S3. Section thickness = 30 μm; scale bars = 2.5 mm. (**D**) Histological examination of distribution in spinal cord under the same experimental conditions. The 2-h time point shows representative lumbar regions, while the 48-h time point shows representative cervical regions (*N* = 3 mice per condition). Section thickness = 30 μm; scale bars = 500 μm. (**E**) Penetration of siRNA from subarachnoid CSF into the parenchyma. Arrow in panel (C) (labeled as “E”) represents the analyzed region. Data are presented from *N* = 3 biological replicates (faint lines are ±standard error of the mean and solid line is the mean). (**F**) Representative image showing perivascular localization of L2-siRNA 48 h after injection. Perivascular space (PVS) is defined as the region between Glut1^+^ endothelium and AQP4^+^ glia limitans. Scale bar = 20 μm (left) or 10 μm (right). The inset is acquired at the indicated area but at a slightly different *z*-plane using confocal microscopy. (**G**) Quantification of perivascular (PV) Cy5 signal between boundaries demarcated by peak Glut1 and Aqp4 intensities. Each dot represents an average of five non-capillary vessels from a single mouse. *N* = 3 biological replicates per condition and data presented as mean ± standard deviation (SD). Statistical significance was calculated using a one-way ANOVA with Bonferroni’s correction (**P< .01, ns – not significant). AU = arbitrary units; AUC = area under curve. Panels (A) (https://BioRender.com/c6ctbqu) (B) (https://BioRender.com/bdbcnj1) were created in BioRender.

To initially examine how the properties of L2-siRNA might impact CSF to brain delivery, we evaluated *in vitro* uptake in neuroblastoma cells and *ex vivo* association with albumin in CSF, with comparisons to unconjugated siRNA (“siRNA”) and cholesterol-conjugated siRNA (“Chol-siRNA”). Structurally, the blunt-ended siRNA design contains alternating 2′OMe and 2′F on both strands in a “zipper” pattern. Phosphorothioate (PS) linkages were used between the last two bases on the ends of both strands, and vinyl phosphonate was integrated at the 5′ antisense position, which is an important feature for maximal silencing in the CNS [8,22]. Structurally, a divalent splitter molecule is placed on the 5′ end of the sense strand, and each arm of the splitter is then linked to a spacer (18 ethylene glycol repeats) terminated by a lipid tail (appending a total of two 18-carbon stearyl groups per siRNA); the 18 ethylene glycol repeats are added in 3 blocks of 6, connected by phosphorothioate bonds (Fig. 1A). There was more cell internalization of L2-siRNA than the parent siRNA, while Chol-siRNA, a benchmark chosen for its known cell penetration capacity [10], had the highest level of uptake in serum-free media (Supplementary Fig. S1A). Despite uptake differences, L2-siRNA carrier-free gene silencing activity was equivalent to Chol-siRNA at 250 nM (Supplementary Fig. S1B).

We hypothesized that a lipid–siRNA conjugate that reversibly binds to albumin would transport effectively throughout the brain because prior studies have shown that albumin-binding dyes have reduced clearance to the periphery after ICV infusion in mice [23] and accumulate in perivascular compartments after ICV injection in rats [24, 25]. We evaluated L2-siRNA association with albumin in *ex vivo* human CSF using FPLC, which showed L2-siRNA eluting in the same fractions as albumin. In comparison, Chol-siRNA and unconjugated siRNA showed no association with albumin (Supplementary Fig. S1C and D). These outcomes suggested that L2-siRNA is a good candidate to potentially leverage albumin for perivascular transport, while also having sufficient capacity for intracellular delivery within the CNS.

### L2-siRNA enters the brain from the subarachnoid and perivascular compartments

Considering the CSF anatomy (Fig. 1B), we investigated how properties of lipid–siRNA conjugates impact CSF to brain transport *in vivo*. We injected mice ICV with Cy5-labeled unconjugated siRNA, Chol-siRNA, or L2-siRNA and examined biodistribution after short and long time points (2 and 48 h). Unconjugated siRNA rapidly dispersed through the mouse brain and spinal cord by 2 h but was minimally retained by 48 h (Fig. 1C and D). Coupled with the observation of high siRNA accumulation in kidneys at 2 h (Supplementary Fig. S2), these results suggest that unconjugated siRNA is not efficiently internalized by brain cells, causing it to pass through and be eliminated quickly from the CNS. In contrast, Chol-siRNA possessed high brain retention at both time points, but the distribution was characterized by steep concentration gradients away from the CSF–brain interface (Fig. 1C). This delivery pattern was also present in the spinal cord, where Chol-siRNA exhibited steep gradients from the subarachnoid space toward the central canal (Fig. 1D). In contrast, L2-siRNA penetrated deeper into the brain from subarachnoid CSF at early time points compared to Chol-siRNA (Fig. 1E). At longer time points, L2-siRNA demonstrated a punctate pattern throughout the brain and spinal cord, presumably associated with perivascular spaces (Fig. 1C and D, and Supplementary Fig. S3).

Leveraging convective CSF flow along perivascular spaces could overcome diffusional limitations and provide a direct mechanism for transport to deep brain structures [13–15]. Localization within the perivascular space was confirmed by the presence of L2-siRNA between endothelial cells (Glut1^+^) and the astrocytic parenchymal glia limitans (Aquaporin-4^+^) (Fig. 1F). Quantifying Cy5 intensity between these markers confirmed that L2-siRNA achieves greater perivascular delivery than Chol-siRNA (Fig. 1G). To evaluate whether delivery to perivascular spaces could be an artifact of the injection volume (i.e. pressure-driven effects), we injected mice ICV with either 2 or 10 μl of L2-siRNA at an equimolar dose. We found that L2-siRNA similarly accessed perivascular spaces in the brain and spinal cord when injected in the lower volume (Supplementary Fig. S4), suggesting that the molecular properties of L2-siRNA are responsible for the observed outcomes. These results collectively indicate that L2-siRNA delivered into the CSF is trafficked through perivascular spaces, providing a conduit for transport into deeper parenchymal structures.

### L2-siRNA achieves durable gene and protein silencing in bulk tissue across multiple brain regions

**Figure 2.**
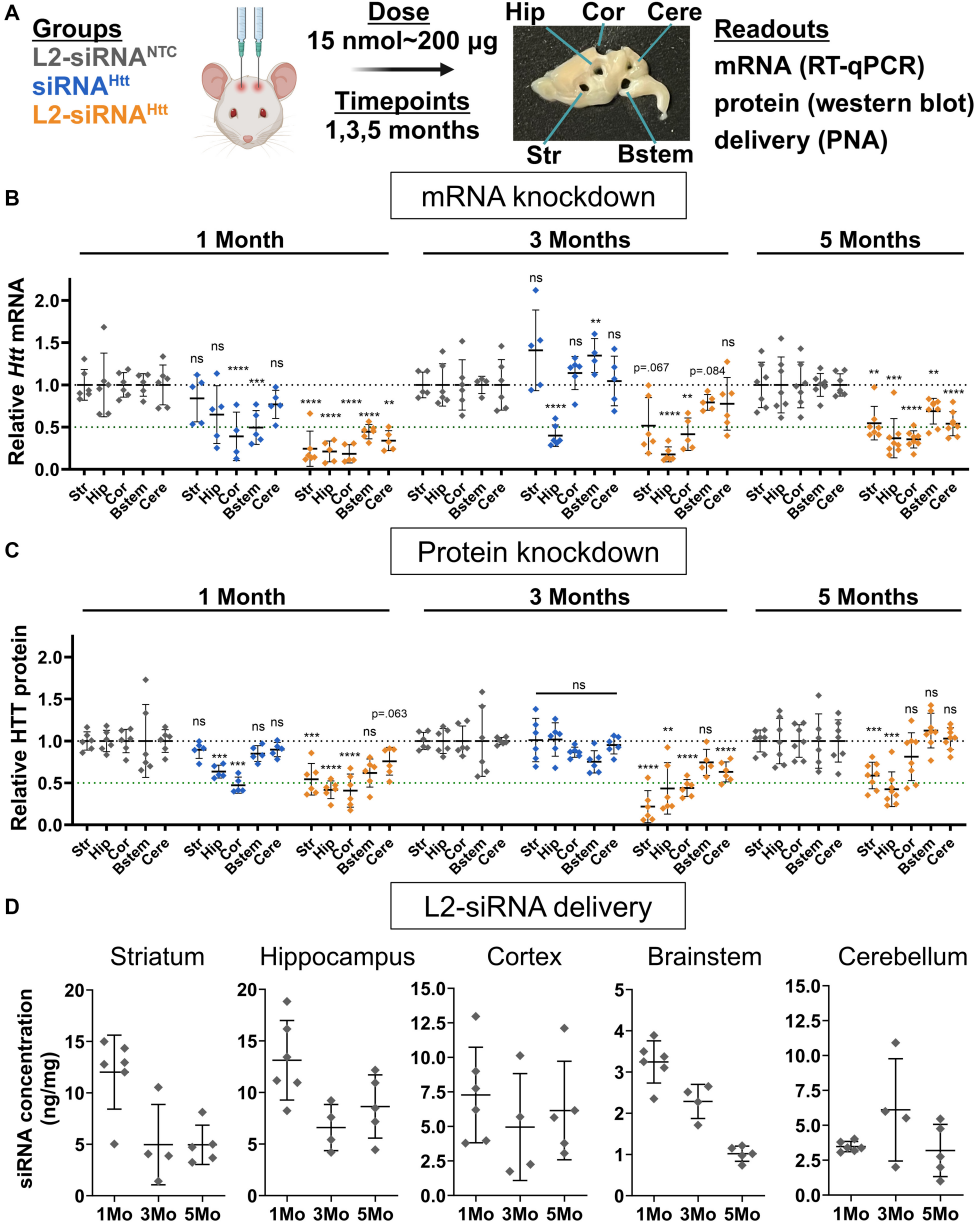
L2-siRNA^Htt^ exhibits potent and sustained gene and protein silencing throughout the parenchyma. (**A**) Experimental approach for evaluating gene silencing. Mice receive a bilateral ICV injection of 15 nmol L2-siRNA^NTC^, siRNA^Htt^, or L2-siRNA^Htt^, and brain tissue is harvested after 1, 3, or 5 months. The brain is sliced into 1-mm sagittal sections, which are biopsy punched (2 mm) to extract different regions for analysis. A representative brain section is shown with biopsy punches extracted. Str, striatum; Hip, hippocampus; Cor, cortex; Bstem, brainstem; Cere, cerebellum. (**B**) *Htt* mRNA levels in parenchymal regions as measured by RT-qPCR. Each region and time point are normalized to its respective L2-siRNA^NTC^ control. For 1 and 3 months, significance was calculated for each region and time point as a one-way ANOVA compared to L2-siRNA^NTC^ with Bonferroni’s correction for multiple comparisons (*N* = 5–6 mice). For 5 months, unpaired two-tailed *t*-tests were performed for each region (*N* = 7–8 mice). (**C**) HTT protein levels in parenchymal regions as measured by western blot. Each region and time point is normalized to its respective L2-siRNA^NTC^ control. Raw western blots are shown in Supplementary Fig. S6. For 1 and 3 months, significance was calculated for each region and time point as a one-way ANOVA compared to L2-siRNA^NTC^ with Bonferroni’s correction for multiple comparisons (*N* = 5–6 mice). For 5 months, an unpaired two-tailed *t*-test was performed for each region (*N* = 7–8 mice). (**D**) Absolute amount of antisense strand (nanograms) per milligram of brain tissue as measured by the PNA assay for L2-siRNA^NTC^. Detailed description of quantification is demonstrated in Supplementary Fig. S7. *N* = 4–6 biological replicates per region per time point (*M* = month). For all panels, data are represented as mean ± SD and each point represents an individual biological replicate (i.e. a single mouse brain). Mo = months. In all panels, statistical significance has the same labels (*P< .05, **P< .01, ***P< .001, ^****^P< .0001, ns – not significant). Panel (A) was created in BioRender (https://BioRender.com/a1zbooy).

Based on the desirable (dispersed yet retained) delivery profile of L2-siRNA, we next assessed the potency and kinetics of target gene silencing. For proof-of-concept studies, we chose *Htt* as a disease-relevant target that is ubiquitously expressed by CNS cells and for which a well-validated siRNA sequence exists (“Htt10150”) [11]. Adult mice were injected ICV with L2-siRNA or an unconjugated siRNA (referred to as siRNA^Htt^) at a 15 nmol dose. We measured *Htt* mRNA by RT-qPCR, HTT protein by western blotting, and absolute amount of antisense strand delivery with a PNA hybridization assay; analyses were performed for CNS regions both proximal to the lateral ventricle injection site (striatum, hippocampus, cortex) and distal to the injection (brainstem, cerebellum) (Fig. 2A). To identify a suitable control, we assessed *Htt* expression in these brain regions after ICV delivery of vehicle (0.9% saline) or a nontargeting siRNA conjugated to the L2 lipid (termed L2-siRNA^NTC^, targeting luciferase, which is not expressed in mice). We determined that treatment with L2-siRNA^NTC^ did not change *Htt* expression appreciably compared to vehicle (Supplementary Fig. S5) and therefore proceeded with L2-siRNA^NTC^ as the primary negative control for gene silencing studies.

At 1 month after injection, *Htt*-targeting L2-siRNA (referred to as L2-siRNA^Htt^) demonstrated potent mRNA knockdown (>50%) in all brain regions tested, whereas siRNA^Htt^ only exhibited silencing in the cortex and brainstem, highlighting the benefit of L2 conjugation (Fig. 2B). Similarly, at the protein level, siRNA^Htt^ generated silencing in the cortex and hippocampus, but less than L2-siRNA^Htt^, which also induced robust knockdown in the striatum (Fig. 2C and Supplementary Fig. S6). At 3 months post-injection, HTT protein returned to baseline in mice receiving siRNA^Htt^, and only the hippocampus exhibited a reduction in *Htt* mRNA, likely because this region is near the injection site. In contrast, mice receiving L2-siRNA^Htt^ exhibited sustained gene and protein knockdown; we note significant reduction in HTT protein at 3 months in the striatum, hippocampus, cortex, and cerebellum. Impressively, at a prolonged 5-month time point, L2-siRNA^Htt^ still achieved mRNA silencing in all examined parenchymal regions. The protein levels were also reduced by L2-siRNA^Htt^ treatment in some regions (striatum, hippocampus), while others returned to basal levels. Unconjugated siRNA^Htt^ was omitted from the 5-month study since its activity was lost at 3 months. Overall, our data show that L2-siRNA^Htt^ potentiates widespread and prolonged gene and protein silencing throughout the CNS compared to the parent siRNA.

To define the relationship between siRNA delivery and gene target knockdown, we quantified L2-siRNA present over a time course using a PNA hybridization assay (Supplementary Fig. S7). Consistently, regions with the highest delivery (striatum, hippocampus, and cortex) also generated the most gene silencing at all time points. While siRNA accumulation decreased after 1 month (as expected), siRNA-L2 was still detectable at 3 and 5 months (Fig. 2D).

### L2-siRNA achieves durable gene and protein silencing throughout the spinal cord

**Figure 3.**
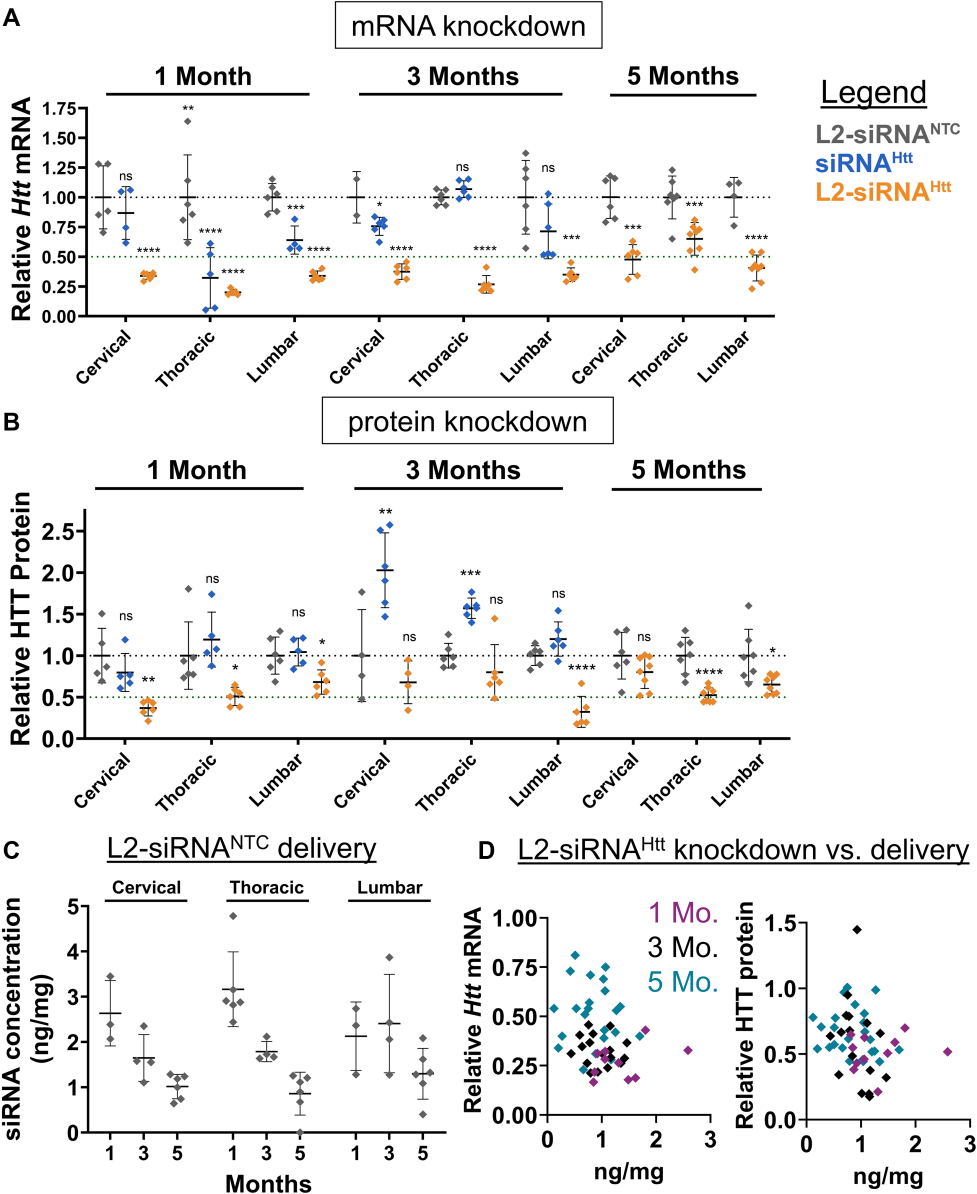
L2-siRNA exhibits potent and sustained mRNA and protein silencing in the spinal cord. (**A**) *Htt* expression levels in spinal cord regions as measured by RT-qPCR. Mice received a bilateral ICV injection (15 nmol total dose) of L2-siRNA^NTC^, siRNA^Htt^, or L2-siRNA^Htt^, and the spinal cords were collected after 1, 3, or 5 months. (**B**) HTT levels in spinal cord regions as measured by western blot. In panels (A) and (B), *N* = 4–6 spinal cords were analyzed at 1 and 3 months, and *N* = 7–8 spinal cord were analyzed at 5 months. For 1 and 3 months, significance was calculated for each region and time point as a one-way ANOVA compared to the L2-siRNA^NTC^ control with Bonferroni’s correction for multiple comparisons. For 5 months, unpaired two-tailed *t*-test was performed for each region. (**C**) Absolute amount of antisense strand (nanograms) per milligram of spinal cord tissue measured by the PNA assay at 1, 3, and 5 months. *N* = 3–6 biological replicates per region per time point. Values below the limit of detection were plotted as 0. (**D**) Relationship between L2-siRNA delivery and gene/protein knockdown. For every graph, each point represents an individual biological replicate (i.e. a single mouse spinal cord) and data are presented as mean ± SD. Mo. = months. In all panels, statistical significance has the same labels (*P< .05, **P< .01, ***P< .001, ^****^P< .0001, ns – not significant).

The spinal cord is directly connected to the CSF circulation and implicated in several diseases druggable with nucleic acid therapies. However, unlike the intrathecal route of administration often used to modulate disease targets of the spinal cord, delivery to the spinal cord requires greater transport from an ICV injection site. Evaluation of the cervical, thoracic, and lumbar spinal cord regions at 1, 3, and 5 months post-injection by the PNA assay showed that L2-siRNA levels in the spinal cord decrease linearly over time from ∼3 to ∼1 ng/mg (Fig. 3C and D). These levels are lower than the parenchyma; however, L2-siRNA^Htt^ gene silencing activity remained high in all segments of the spinal cord at both the mRNA and protein levels, whereas unconjugated siRNA did not mediate knockdown of HTT protein (Fig. 3A and B). These outcomes further highlight the effectiveness of L2-siRNA throughout the CNS.

### L2-siRNA effectively targets diverse CNS cell types

Having characterized delivery and knockdown in bulk tissue, our next goal was to develop an understanding of L2-siRNA uptake and activity in specific cell types. To assess cell type-dependent uptake, mice were administered Cy5-tagged L2-siRNA or unconjugated siRNA (10 nmol) by ICV, and flow cytometry was performed after 48 h to measure Cy5 levels in various cell types defined as Thy1^+^ neurons, ACSA2^+^ astrocytes, CD11b^+^ microglia/macrophages, and CD31^+^ endothelial cells. Oligodendrocytes were removed from analysis based on O1^+^ staining, as myelin can promote nonspecific antibody binding [26]. We found that total and cell-specific uptake were increased by L2 conjugation (Supplementary Fig. S8), in agreement with our prior *in vitro* data (Supplementary Fig. S1A). L2-siRNA uptake into neurons was the lowest out of the cells examined, but this may have been due to known challenges in recovering neurons after dissociation (<2% of total population). Microglia, which are resident CNS phagocytes, showed the highest L2-siRNA uptake (Supplementary Fig. S8).

**Figure 4.**
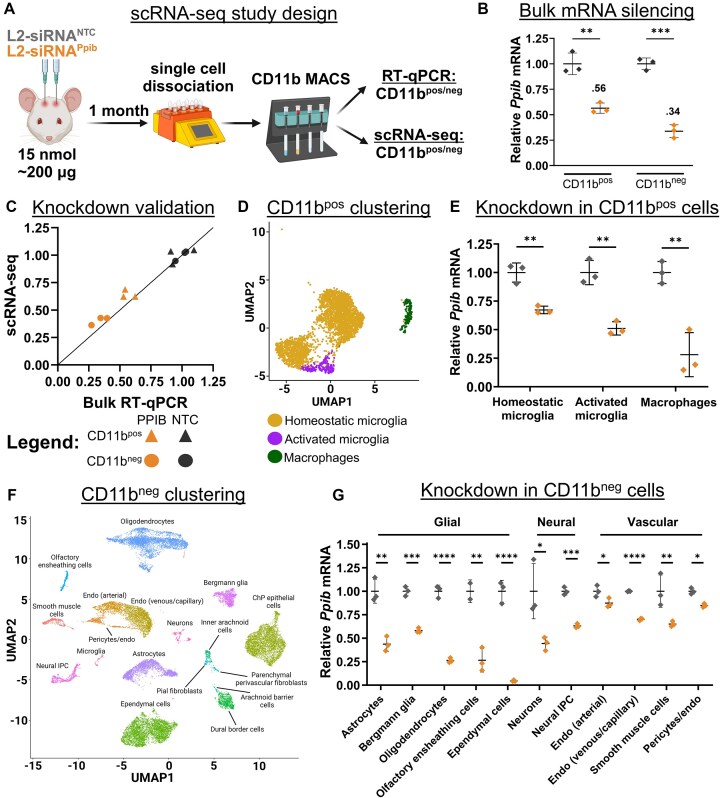
L2-siRNA potentiates gene silencing across diverse parenchymal cells. (**A**) Experimental design to assess cell-specific gene silencing. Mice were administered 15 nmol of L2-siRNA^Ppib^ or L2-siRNA^NTC^, and after 1 month, brains were dissociated into single cells and bead-sorted into Cd11b^pos^ and Cd11b^neg^ populations, which were further separated for scRNA-seq or RT-qPCR. For all experiments in this figure, *N* = 3 individual mice were analyzed (biological replicates) for both L2-siRNA^Ppib^ and L2-siRNA^NTC^. (**B**) *Ppib* expression in Cd11b^pos^ and Cd11b^neg^ populations measured by RT-qPCR. Statistical significance was calculated using an unpaired two-tailed *t*-test. (**C**) Comparison of *Ppib* expression between bulk RT-qPCR (*x*-axis) and scRNA-seq averaged across all identified cells (*y*-axis). Line with a slope of 1 represents equivalent expression between readouts. (**D**) UMAP plot showing clustering and annotation of Cd11b^pos^ populations. (**E**) Average *Ppib* expression across different Cd11b^pos^ cell types, normalized to L2-siRNA^NTC^. (**F**) UMAP dimension reduction plot showing clustering and annotation of Cd11b^neg^ populations. Unless indicated with a line, all text labels lie above their respective cluster. (**G**) Average *Ppib* expression across different Cd11b^neg^ cell types, normalized to L2-siRNA^NTC^. Statistical significance was calculated using an unpaired two-tailed *t*-test for all cell types (*P< .05, **P< .01, ***P< .001, ^****^P< .0001, ns – not significant). Data are presented as mean ± SD in every panel. Panels (A) (https://BioRender.com/dnyh4zw), (D) (https://biorender.com/obsa3f3), and (F) (https://biorender.com/hci4ojo) were created using BioRender.

To determine whether L2-siRNA is active in disease-relevant cell types, we developed an scRNA-seq strategy to measure gene silencing in heterogeneous cell populations throughout the brain. Here, we used an siRNA sequence targeting *Ppib* because this gene is abundantly and ubiquitously expressed across CNS cell types [27]. Potent siRNA sequences had also been designed and validated against *Ppib* elsewhere [28]. Matching the experimental parameters of bulk tissue knockdown analyses, we injected 15 nmol of either L2-siRNA*^Ppib^* or L2-siRNA^NTC^ and collected cells after 1 month for evaluation. Since we observed substantial uptake into Cd11b^+^ cells by flow cytometry, we elected to sort out and enrich myeloid cells, yielding two datasets: Cd11b^pos^ cells encompassing microglia and macrophages, and Cd11b^neg^ cells comprising all other isolated meningeal and parenchymal populations (Fig. 4A). To verify gene silencing with orthogonal methodology, cells not utilized for scRNA-seq were analyzed using RT-qPCR, which confirmed gene silencing in both Cd11b^pos^ and Cd11b^neg^ populations with an average of 45% and 65% *Ppib* knockdown, respectively (Fig. 4B). We compared *Ppib* knockdown measured by scRNA-seq and RT-qPCR and these outputs closely matched for both CD11b^pos^ and CD11b^neg^ populations (Fig. 4C), providing confidence in the accuracy of measuring cell-specific gene silencing with scRNA-seq.

To determine gene silencing in myeloid cells, Cd11b^pos^ cells were annotated and clustered into three populations using standard markers (Fig. 4D). Homeostatic microglia are characterized by expression of *Crybb1*, *Cst*3, *P2ry12*, *Pros1*, and *Tmem119*, while activated microglia additionally express *Apoe*, *Spp1*, *Lpl*, and *Lyz2* (Supplementary Fig. S9A and B) [29, 30]. CNS macrophages include perivascular, meningeal, and choroid plexus macrophage populations, which are enriched in *Pf4*, *Lyz2*, *Ms4a7*, *Ccl24*, and *F13a1*; this population constituted ∼6% of Cd11b^pos^ cells (Supplementary Fig. S9C). Compared to the nontargeting control, L2-siRNA*^Ppib^* potentiated gene silencing in homeostatic (∼40%) and activated microglia (∼50%), as well as resident CNS macrophages (∼70%) (Fig. 4E and Supplementary Fig. S9D). Microglia are notoriously refractory to gene silencing, highlighting the significance of this result [31].

In addition to potent myeloid gene knockdown, L2-siRNA*^Ppib^* was active in a variety of other cell types identified in the Cd11b^neg^ population (Fig. 4F and Supplementary Fig. S10). For example, glial cells such as astrocytes and oligodendrocytes exhibited considerable *Ppib* knockdown, as well as ependymal cells, which line the ventricles and directly interface with CSF (Fig. 4G). Neurons experienced modest gene silencing, but as expected, we could not subcluster into neuron subtypes due to the low capture of this population after dissociation. Endothelial cells were subclustered into venous/capillary and arterial populations (Fig. 4F); there was low but significant knockdown in arterial endothelial cells, and slightly higher silencing in the venous/capillary cells (Fig. 4G). Modest gene silencing activity in these cells is consistent with low intensity of uptake observed by flow cytometry and is consistent with the anticipated results following CSF delivery. Overall, we established a novel approach for detecting cell-specific siRNA silencing using scRNA-seq. We found that L2-siRNA*^Ppib^* exhibits broad gene silencing activity that varied in magnitude across diverse CNS cell types.

### L2-siRNA effectively targets brain border cells

**Figure 5.**
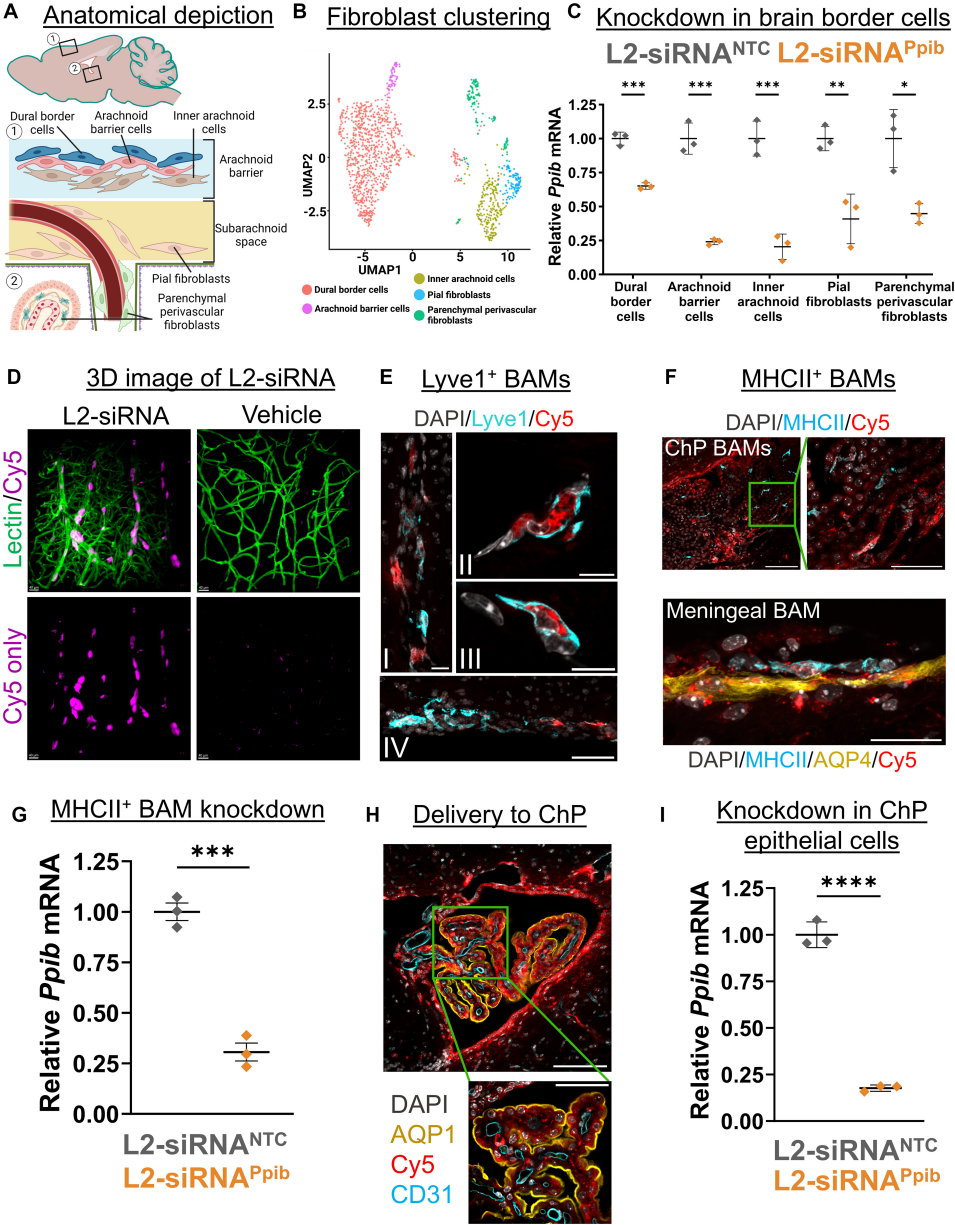
L2-siRNA exhibits effective targeting and gene silencing in brain border cells. (**A**) Anatomical location of fibroblasts in the leptomeninges. (**B**) UMAP dimension reduction plot showing sub-clustering of fibroblast populations. All fibroblasts outside the meninges are referred to as “parenchymal perivascular fibroblasts,” which include perivascular, choroid plexus, and potentially median eminence fibroblasts. (**C**) scRNA-seq assessment of *Ppib* knockdown in fibroblasts of the brain borders at a 1-month time point. Knockdown is normalized to L2-siRNA^NTC^. (**D**) Three-dimensional image demonstrating extensive perivascular delivery of L2-siRNA around vessels in the cortical surface (48 h, 10 nmol dose). Representative images displayed are consistent across replicates. (**E**) Representative image of Cy5-tagged L2-siRNA delivery to Lyve1^+^ BAMs 48 h after injection. (I) Delivery along penetrating vessel; scale bar = 20 μm. (II, III) Subcellular localization in perivascular Lyve1^+^ BAMs; scale bar = 10 μm. (IV) Delivery to ventral meningeal Lyve1^+^ BAMs; scale bar = 50 μm. (**F**) Representative images of Cy5-tagged L2-siRNA delivery to MHCII^+^ BAMs 48 h after injection. Scale bar = 100 μm (top left), 50 μm (top right); 25 μm (bottom). (**G**) scRNA-seq assessment of *Ppib* knockdown in MHCII^+^ macrophages at a 1-month time point. Knockdown is normalized to L2-siRNA^NTC^. (**H**) Representative image of Cy5-tagged L2-siRNA delivery to cells of the fourth ventricle choroid plexus (ChP) 48 h after injection (2 nmol in 10 μl ICV). Aqp1 marks the apical side of ChP epithelial cells. Scale bar = 100 μm (top) and 50 μm (bottom). (**I**) scRNA-seq assessment of *Ppib* knockdown in ChP epithelial cells at a 1-month time point. Knockdown is normalized to L2-siRNA^NTC^. For panels (C), (G), and (I), *N* = 3 individual mice (biological replicates) for both L2-siRNA*^Ppib^* and L2-siRNA^NTC^. Data are presented as mean ± SD for all graphs. Statistical significance was calculated using an unpaired two-tailed *t*-test (*P< .05, **P< .01, ***P< .001, ^****^P< .0001). Panels (A) (https://biorender.com/3ynqsts) and (B) (https://BioRender.com/czhlce8) were created using BioRender.

Recent advances in single-cell biology have revealed the diversity of cells present at brain borders, including the choroid plexus and meninges, as well as the previously discussed perivascular spaces. There is a growing appreciation for the role brain borders play in disease [32], but no prior studies have rigorously evaluated siRNA delivery to the cells residing in these borders. Since brain borders directly interface with CSF, we investigated L2-siRNA-mediated gene silencing in these compartments. First, we examined gene silencing in leptomeningeal cells after ICV delivery. Recent characterization of fibroblast-like cells in the meninges identified five transcriptionally and spatially unique cell populations [33]. Anatomically, the arachnoid membrane separates the dura from subarachnoid CSF; from top to bottom, this membrane is composed of dural border cells, arachnoid barrier cells, and inner arachnoid cells. Beneath this membrane are pial fibroblasts, either associated with vessels (perivascular) or sitting on the pial membrane (Fig. 5A). We re-clustered our scRNA-seq data set to identify the aforementioned cells (Fig. 5B). Impressively, knockdown was observed in all fibroblast-like populations, with dural border cells experiencing the least gene silencing, possibly because of their location on the opposing side of the arachnoid membrane. The most potent knockdown was observed in cells directly contacting CSF, such as arachnoid barrier cells, pial fibroblasts, and arachnoid barrier cells (Fig. 5C).

Border-associated macrophages also reside in CSF outside of the brain parenchyma, primarily in the choroid plexus, meninges, and perivascular spaces, and are central players both in maintaining homeostasis and mediating CNS inflammatory responses [34, 35]. We visualized the vascular network in three dimensions using CLARITY and light-sheet microscopy and observed a high concentration of L2-siRNA within perivascular cells throughout the parenchyma (Fig. 5D). To assess the identity of these perivascular cells, we stained for macrophages based on recent work that subdivided border-associated macrophages into MHCII^+^ macrophages, which express genes involved in antigen presentation, and Lyve1^+^ scavenger macrophages, which perform a variety of essential functions, including the regulation of CSF flow dynamics [36]. We observed extensive delivery to perivascular Lyve1^+^ macrophages, as evidenced by localization of bright L2-siRNA puncta (Fig. 5E). MHCII^+^ macrophages are most abundant in the choroid plexus and meninges [37], and we similarly observe widespread uptake of L2-siRNA in this population (Fig. 5F). To assess knockdown in macrophages, we subclustered these populations from the L2-siRNA*^Ppib^* scRNA-seq knockdown study (Supplementary Fig. S11). While the Lyve1^+^ population was too sparse to evaluate, we found robust gene silencing in the MHCII^+^ macrophages (Fig. 5G). Lastly, we examined delivery to other cells in the choroid plexus and noted a high level of delivery to choroid plexus epithelial cells corresponding to robust gene silencing (Fig. 5H and I).

### L2-siRNA does not induce hallmarks of toxicity

Treatment with L2-siRNA was overall well tolerated in rodents. Recent studies suggest that nucleic acid drugs delivered in high concentrations into the CSF can sequester divalent cations leading to seizures or “acute neuronal activation response” [38, 39]. Yet, we never observed this phenomenon in mice or rats with our formulations. In addition, every mouse injected with an L2-siRNA conjugate (Htt, Ppib, NTC) survived to its predetermined endpoint.

We also searched for potential adverse reactions, including reactive astrogliosis, neuroinflammation, and systemic organ damage. Astrogliosis is a multi-faceted process through which astrocytes respond to damage or disease and is characterized by elevated GFAP expression, indicative of cytoskeletal hypertrophy [40]. Immunohistochemical staining of GFAP 1 month after ICV injection did not show any differences between L2-siRNA groups (NTC, Htt) and vehicle (Supplementary Fig. S12A and B). These results are consistent with western blot quantification of GFAP in the hippocampus (Supplementary Fig. 12C), collectively suggesting that L2-siRNA does not induce astrogliosis.

Next, we investigated whether ICV injection of L2-siRNA induced any neuroinflammatory responses. Microglia activation was assessed by Iba1 expression, indicative of a proliferative phenotype [41]. Iba1 protein levels were unchanged after L2-siRNA injection compared to vehicle (Supplementary Fig. S12D and E). To more broadly examine CNS inflammation, we utilized an ELISA cytokine panel to evaluate cortical and striatal tissue 2 weeks after ICV injection, at either the standard dose used in knockdown studies (15 nmol) or a lower dose (5 nmol). Notably, no difference in pro-inflammatory cytokine, chemokine, or growth factor levels was observed between vehicle and L2-siRNA^Htt^ (Supplementary Figs S12F and S13). Consistent with prior studies showing no evidence of systemic toxicity induced by L2-siRNA after intravenous injection [12,42], we similarly did not observe changes in serum markers after ICV injection in mice or intrathecal injection in rats (Supplementary Figs S14 and S15). Collectively, L2-siRNA did not exhibit any detectable toxicities at the doses utilized to achieve potent and durable target gene silencing, suggesting that L2-siRNA has a practical therapeutic index.

### L2-siRNA is distributed throughout the rat brain and silences *Htt* in diverse CNS regions after intrathecal delivery

**Figure 6.**
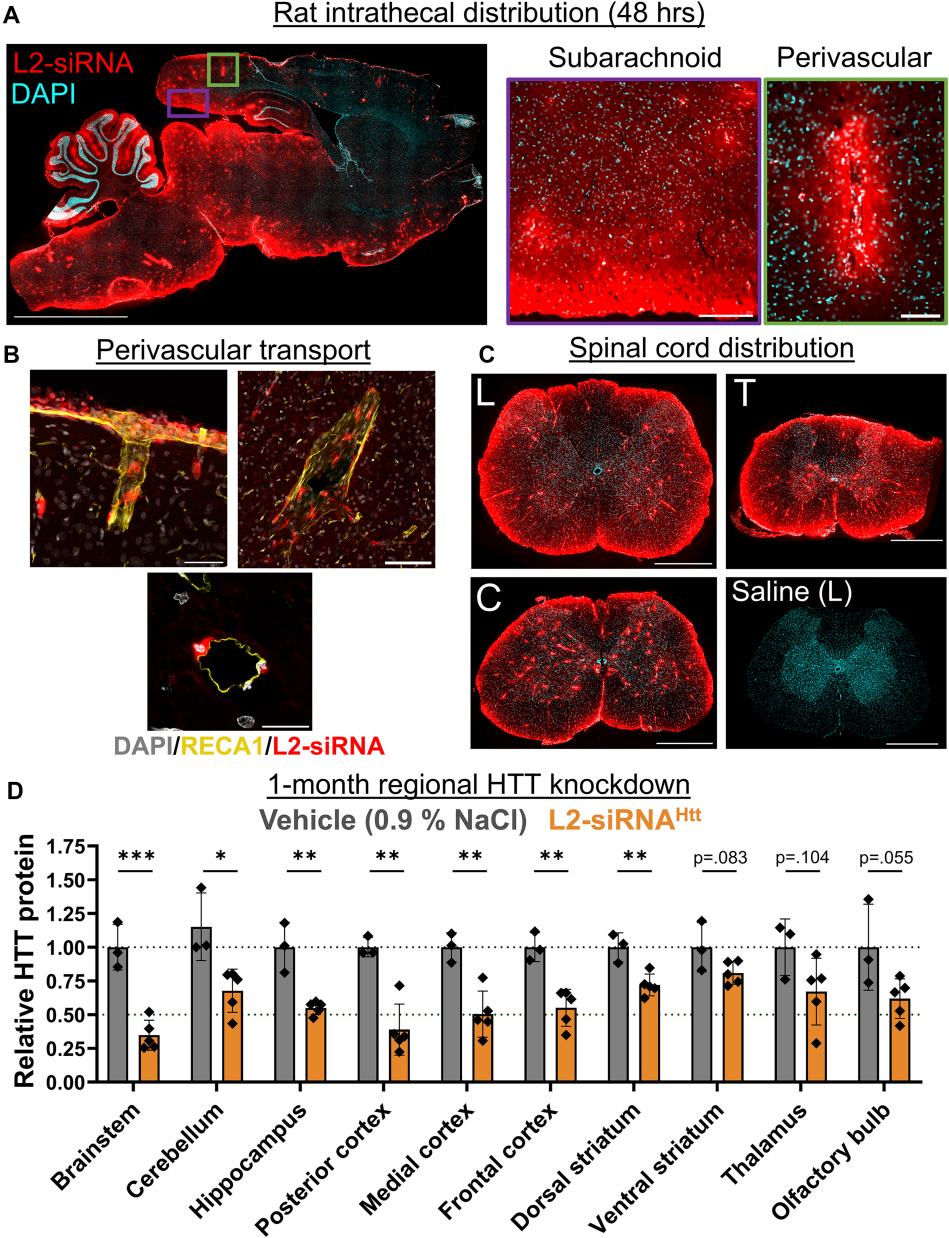
Intrathecal delivery of L2-siRNA in rats achieves broad CNS distribution and *Htt* knockdown. (**A**) Distribution of Cy5-labeled L2-siRNA (400 μg, ∼30 nmol) throughout the parenchyma 48 h after intrathecal injection in rats. Scale bar = 5 mm; section thickness = 15 μm. Zoomed areas highlight delivery from subarachnoid space into the posterior cortex (left, scale bar = 200 μm) as well as perivascular delivery and transport into the surrounding cells (right, scale bar = 100 μm). (**B**) To more definitely examine perivascular transport, vessels were stained with RECA-1. Perivascular transport is observed at the meningeal surface, penetrating the parenchyma (top left image, scale bar = 50 μm). The transport continues to deeper vessels, as demonstrated by a representative thalamic vessel (top right, scale bar = 100 μm). L2-siRNA can also be visualized in cells from a vessel cross section (bottom image, scale bar = 20 μm). (**C**) Representative images of L2-siRNA delivery throughout the lumbar (“L”), thoracic (“T”), and cervical (“C”) regions of the spinal cord. A saline-injected rat is provided as a reference. Section thickness = 15 μm; scale bar = 1 mm. (**D**) Regional HTT knockdown assessed 1 month after intrathecal injection of L2-siRNA^Htt^ (800 μg, ∼60 nmol). Each point represents an individual biological replicate (i.e. a single rat) and data are presented as mean ± SD, *N* = 3–5. Statistical significance is calculated with unpaired two-tailed *t*-tests for each region (*P< .05, **P< .01, ***P< .001).

The CSF has several access points for drug delivery, the most common therapeutic route being intrathecal administration into the lumbar cistern. To more closely mimic the clinical standard [43], we assessed biodistribution and knockdown after intrathecal administration in rats. We administered a bolus dose of Cy5-labeled L2-siRNA (30 nmol, ∼400 μg) through a catheter inserted via lumbar puncture into the cauda equina space [17]. After 48 h, we performed histology of the entire rat brain, which showed that L2-siRNA was transported along the caudal–rostral neuroaxis, where it penetrated the parenchyma from subarachnoid and perivascular CSF pathways (Fig. 6A). To further confirm perivascular localization, we stained vessels with rat endothelial cell antibody (RECA1), which revealed considerable transport along penetrating vessels both at the surface and in deep regions of the brain (Fig. 6B). Robust distribution throughout all regions of the spinal cord was observed, which is expected for intrathecal delivery based on proximity to the injection site (Fig. 6C).

We next assessed longer-term delivery and gene silencing of L2-siRNA^Htt^ (60 nmol, ∼800 μg) compared to a vehicle control (0.9% NaCl). We standardized an approach to micro-dissecting the rat brain for delivery and knockdown assessments (Supplementary Fig. S16). The PNA assay revealed differential levels of regional siRNA accumulation; the cerebellum, brainstem, and olfactory bulb exhibited the greatest retention of oligonucleotide, while delivery to deep brain structures (striatum and thalamus) was ∼2–3× lower than delivery to the cortex (Supplementary Fig. S17A–C). In terms of *Htt* mRNA reduction, L2-siRNA^Htt^ generated particularly potent gene silencing activity throughout the cortex, hippocampus, brainstem, olfactory bulb, cerebellum, and ventral striatum (Supplementary Fig. S17D). Impressively, HTT protein levels were lowered in all brain regions examined but were attenuated in the striatum and thalamus (∼25% lowering) compared to the cortical regions (Fig. 6D). As expected for an intrathecal injection, delivery and gene inhibition were effective throughout the spinal cord as well (Supplementary Fig. S18 and S19).

### L2-siRNA benchmarking versus ASO

**Figure 7.**
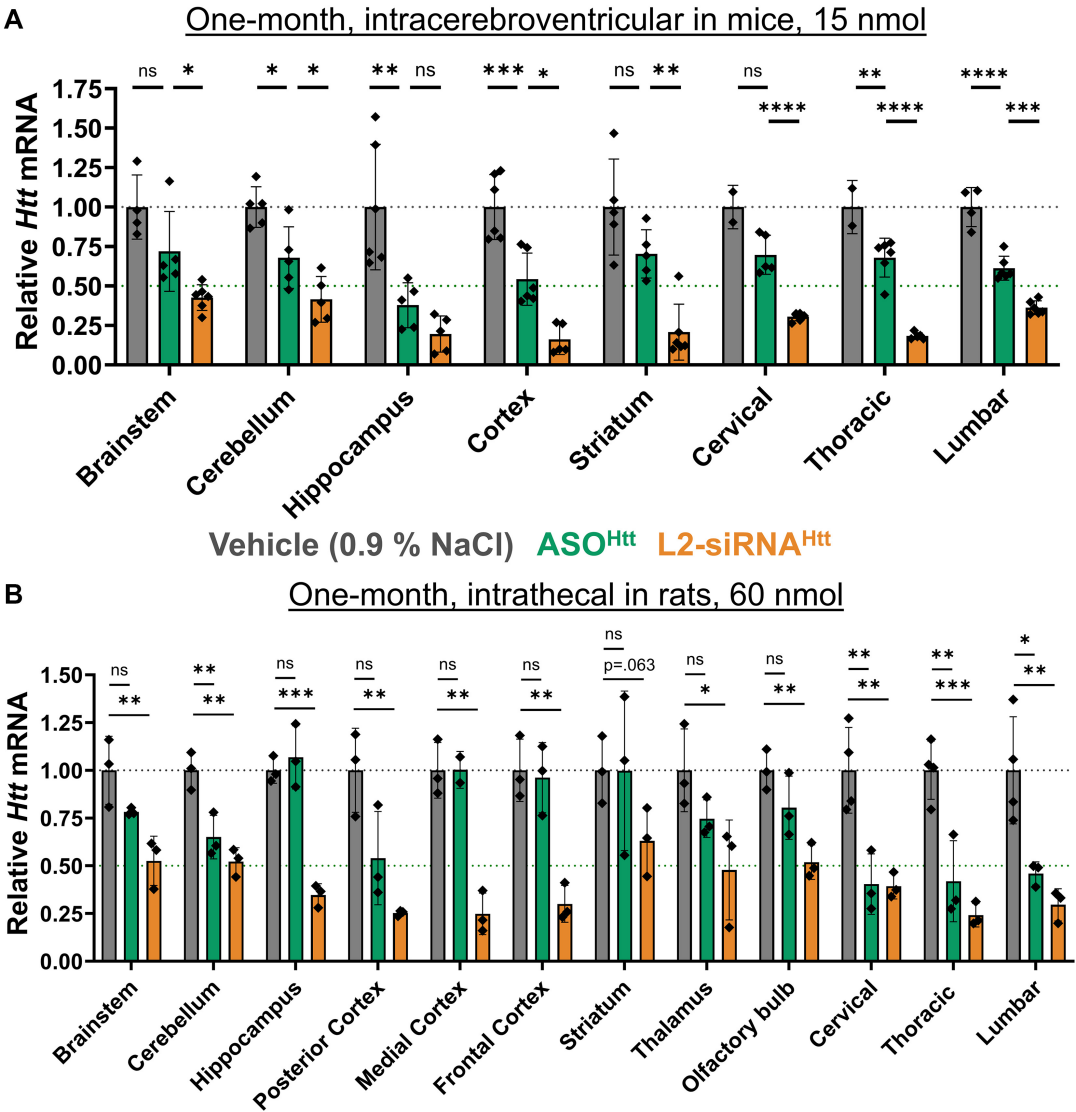
Comparing Htt lowering between L2-siRNA^Htt^ and ASO^Htt^. (**A**) Mice were administered 15 nmol of ASO^Htt^ or L2-siRNA^Htt^ ICV and knockdown was normalized to a vehicle (0.9% NaCl) control. *Htt* levels were determined by RT-qPCR 1 month after injection. Since the ASO^Htt^ and L2-siRNA^Htt^ knockdown studies were performed on different cohorts, each sample is normalized to their respective cohort of vehicle controls, and only the vehicle from the ASO^Htt^ study is plotted here (*N* = 6 mice per group). One-way ANOVA was performed for each region comparing ASO^Htt^ to vehicle and L2-siRNA^Htt^ to ASO^Htt^ (Bonferroni’s correction for multiple comparisons). (**B**) Assessment of gene silencing 1 month after intrathecal delivery normalized to vehicle control. One-way ANOVA was performed for each region comparing ASO^Htt^ and L2-siRNA^Htt^ to vehicle (Bonferroni’s correction). Each point represents a rat (*N* = 3–4) and bars represent mean ± SD (*P< .05, **P< .01, ***P< .001, ^****^P< .0001, ns – not significant).

We also compared L2-siRNA^Htt^ to an *Htt*-targeting ASO in both mice and rats. To match dosing conditions to prior L2-siRNA^Htt^ studies, we administered an equimolar dose (15 nmol for ICV and 60 nmol for intrathecal) of the “MoHu” gapmer ASO^Htt^ that was designed with homology for mouse, rat, and human *HTT* [16]. Gene silencing was assessed at a 1-month time point, and given the disparate structures between the two molecules, normalization was performed relative to a vehicle control. We found that L2-siRNA^Htt^ was more potent than ASO^Htt^ in these experimental conditions, demonstrating statistically significant enhancement of mRNA knockdown in all parenchymal and spinal cord regions except the hippocampus, where both compounds caused robust knockdown (Fig. 7A). Of particular note, L2-siRNA^Htt^ was more effective in the striatum (∼80% reduction) than ASO^Htt^ (∼25% reduction) in mice, which is highly relevant considering the striatum is the region predominantly affected by HD. Similarly, after intrathecal injection into rats, L2-siRNA^Htt^ was more potent than ASO^Htt^ in all regions examined, most notably in regions distal to the injection site, such as the frontal cortex, thalamus, striatum, and olfactory bulb (Fig. 7B). Collectively, this evidence showcases the potential of using L2-siRNA to achieve broad gene silencing.

## Discussion

A key objective in developing disease-modifying therapies for neurodegenerative disorders is delivering molecularly targeted inhibitors to specific neuroanatomical sites. Along these lines, the central translational challenge for nucleic acid therapies is achieving gene silencing throughout the brain. Here, we showed that L2-siRNA achieves a balanced cell internalization profile, distributing further and more homogeneously into the parenchyma from subarachnoid and perivascular CSF compared to Chol-siRNA. Rigorous characterization of L2-siRNA activity in mice and rats revealed durable silencing throughout numerous regions and cell types of the CNS and brain borders. In this discussion, we provide additional commentary on route-specific gene silencing, considerations for assessing cell-specific knockdown, and ASO versus siRNA comparisons.

L2-siRNA achieved sustained gene and protein inhibition after ICV injection in mice. Notably, L2-siRNA accumulation and huntingtin lowering were detectable 5 months after a single injection. These observations are consistent with the recently posited endolysosomal depot phenomenon, which suggests that once delivered, chemically stabilized siRNA remains intact and slowly escapes from sequestration within intracellular endolysosomal vesicles, gaining access to the cytoplasmic gene silencing machinery; this phenomenon allows the internalized siRNA to gradually achieve intracellular bioavailability over time, a feature that is advantageous for achieving durable gene silencing bioactivity and minimizing treatment frequency in chronic diseases [44]. To more precisely understand the kinetics of L2-siRNA gene silencing activity, we measured both mRNA and protein at 1, 3, and 5 months. We observed that while mRNA knockdown was most potent at 1 month, protein knockdown peaked at 3 months, presumably owing to the long half-life of the HTT protein (∼2 weeks). We also noticed that mRNA lowering was consistently more effective than protein knockdown. Yet, other groups have reported the opposite trend, demonstrating that nuclear *Htt* mRNA, which is abundant in neurons, remains inaccessible to siRNA-mediated degradation [22,45]. We believe this discrepancy could be explained by methodological differences, whereby the RNA extractions we performed favor isolation of cytoplasmic over nuclear transcripts.

We also showed that CSF access points for oligonucleotide injections can change biodistribution [46]. While intrathecal delivery is the most common oligonucleotide delivery route in humans, most screening studies in mice are conducted using the ICV route due to technical challenges associated with intrathecal injections in smaller rodents. As demonstrated in this manuscript, we believe that the ICV mouse model is a great tool for studying general mechanisms of CSF to brain transport (i.e. access to subarachnoid and perivascular pathways), on-target gene silencing kinetics, and cell-specific activity. Importantly, however, ICV delivery does not recapitulate the regional distribution observed after an intrathecal injection, especially in deep brain structures. Unlike therapeutics injected intrathecally, oligonucleotides administered ICV can cross the ependymal lining of the ventricles and achieve robust delivery to deep brain regions such as the striatum. In contrast, intrathecally administered compounds must travel further along the spinal cord before reaching rostral brain regions [47]. Indeed, while we observed widespread L2-siRNA transport and knockdown in all regions examined after rat intrathecal injection, we noted an attenuation in gene silencing activity in distal structures (thalamus, striatum) compared to regions proximal to the injection site (spinal cord, brainstem, posterior cortex). Overall, we emphasize how we used the mouse ICV and rat intrathecal models to rigorously characterize L2-siRNA transport and gene silencing properties.

Disease-associated genes are usually expressed by many CNS cell types, but select populations play an outsized role in driving disease progression [48]. To determine suitable applications for L2-siRNA technology, we established a cell type-specific atlas of L2-siRNA gene silencing activity. While single-nucleus atlases have generated informative datasets for ASOs, this method is not suitable for siRNAs, which only degrade cytoplasmic mRNA [49]. As such, we developed an approach to assess L2-siRNA cell type-specific knockdown using scRNA-seq. In designing this study, the first key consideration was choosing a gene target. Because sequencing coverage results in some dropout (apparent zeroes) for each gene, scRNA-seq is conventionally used to characterize cell types and pathways, processes governed by changes in multiple transcripts [50]. To minimize the proportion of dropouts, it was essential to choose a highly abundant target transcript like *Ppib*. The second consideration we encountered was deciding how to capture rare populations. To enrich border-associated macrophages, we elected to sort CD11b-positive cells. Yet, as sequencing costs have declined, we would instead recommend future studies consider sequencing a greater number of unsorted cells compared to the approach of pre-sorting for a population of interest, which biases cell capture.

The scRNA-seq study revealed new insights into L2-siRNA activity that could not be determined from bulk tissue analyses. We validated the methodology by running RT-qPCR on the same samples and confirmed that the measured knockdown was equivalent to the scRNA-seq computation. Further support for the reliability of this approach came from biological interpretation of knockdown in different cell types. For example, ependymal cells line the ventricles and experience the highest concentration of siRNA after an ICV injection; we found that these cells experienced the greatest gene silencing. Another key finding was that microglia (activated and homeostatic), an important but difficult to manipulate cell type, could be effectively targeted with L2-siRNA. Overall, this cell-specific investigation showed us that L2-siRNA is effective in most CNS cell types, particularly those near CSF flow.

With the advent of different classes of biotechnologies capable of precisely modulating gene targets, it has become imperative to benchmark these tools against one another to determine which would be most effective for treating specific diseases. Notably, several ASO therapies targeting genes in the spinal cord provide clinical benefit after intrathecal administration; Spinraza and Tofersen have extended survival for patients with spinal muscular atrophy and amyotrophic lateral sclerosis, respectively [43,51]. While effective in regions proximal to the lumbar injection site, numerous studies suggest that ASOs are not well suited for deep brain structure targets [16,47,52]. In a direct comparison, we demonstrated that L2-siRNA^Htt^ was more potent than ASO^Htt^ across all brain regions in the intrathecal rat model, including the striatum where HD pathology manifests. These differences could reflect structural differences, as siRNAs form a linear duplex and diffuse effectively through the narrow extracellular spaces of the brain [53]. In contrast, single-stranded ASOs form a less rigid and more globular structure and may exhibit nonspecific protein association, thereby limiting deep brain penetration. In addition, siRNAs are more potent on a per-molecule basis compared to ASO; ∼2000 cytoplasmic siRNAs are estimated to achieve maximal silencing, whereas ∼50 000 are required for an ASO [54, 55]. One advantage ASOs do possess over siRNA is their modularity—they can be designed to modulate splicing, degrade mRNA, or even inhibit miRNAs and therefore constitute an important tool in the arsenal of therapies for treating neurodegenerative diseases [56].

Overall, this study examined properties of lipid–siRNA conjugates that facilitate CSF to brain delivery. We performed detailed characterization of L2-siRNA transport, bulk tissue gene silencing, cell type-specific activity, and toxicity. Collectively, these data suggest that L2-siRNA possesses unique properties that enhance its transport and brain-wide distribution relative to other conjugates, thus yielding a promising platform technology for silencing genes implicated in CNS disorders.

## Supplementary Material

gkaf600_Supplemental_File

## Data Availability

Raw data and processed Seurat objects are available at ArrayExpress under accession number E-MTAB-13964. Code for reproducing the single-cell RNA sequencing analysis has been deposited as an interactive dataset in the Broad Institute single-cell portal (https://singlecell.broadinstitute.org/single_cell/study/SCP2964/cell-specific-gene-silencingof-lipid-sirna-conjugates-in-the-mouse-central-nervous-system-cns). The single-cell code has also been deposited at Zenodo: https://zenodo.org/records/15257645.
