# Supplementary material for: Elucidating brain transport pathways and cell type-dependent gene silencing of a durable lipid–siRNA conjugate administered into cerebrospinal fluid
Source: Nucleic Acids Res. 2025 Jul 2;53(12):gkaf600. doi: 10.1093/nar/gkaf600 (PMC12214009; doi:10.1093/nar/gkaf600)
Supplement: gkaf600_Supplemental_File [file gkaf600_supplemental_file.pdf]

Supplementary Data for

Elucidating brain transport pathways and cell type-dependent gene silencing of a durable lipid-siRNA conjugate administered into cerebrospinal fluid

Alexander G. Sorets *et al.*

*Corresponding author. Ethan S. Lippmann (ethan.s.lippmann@vanderbilt.edu) or Craig L. Duvall (craig.duvall@vanderbilt.edu)

This PDF file includes:

Supplementary Figures S1 to S19
Table S1

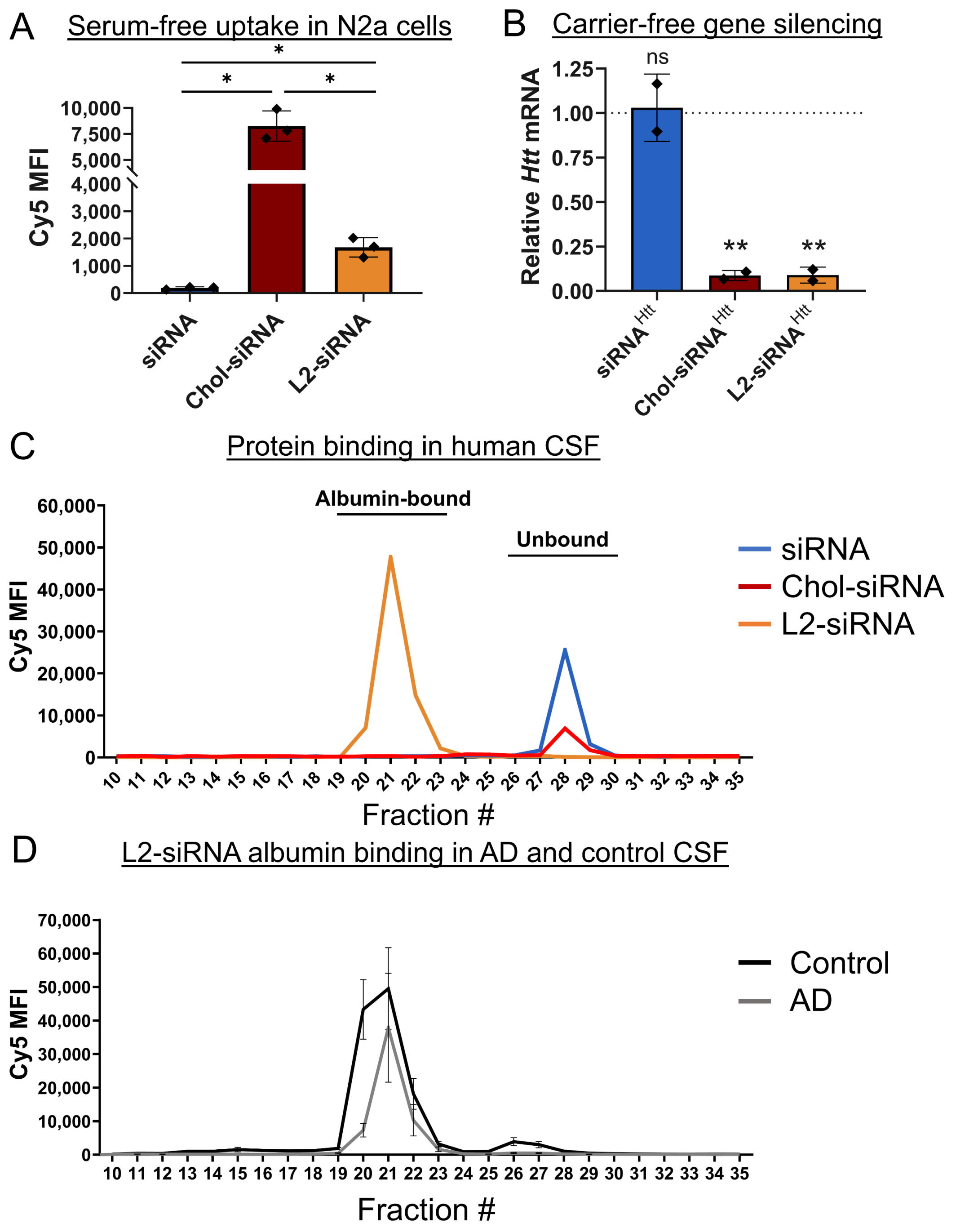

Supplementary Figure S1. Physicochemical properties of siRNA conjugates

A. Cell uptake was evaluated by flow cytometry in N2a cells after a 2-hour incubation with unconjugated siRNA, Chol-siRNA, or L2-siRNA (60 nM). Mean fluorescence intensity (MFI) is reported from three independent experiments, where the bars represent mean ± SD. One-way ANOVA without assuming equal SD was performed with Dunnett's T3 correction for multiple comparisons (*p<0.05).

B. Carrier-free *Htt* knockdown in N2a cells assessed by RT-qPCR after a 48-hour incubation with siRNA^{Htt}, Chol-siRNA^{Htt}, or L2-siRNA^{Htt} normalized to L2-siRNA^{NTC} (represented by dotted line

at Y=1.0). Data reported from two independent experiments, each normalized to L2-siRNA^{NTC}, with bars representing mean ± SD. One-way ANOVA compared to L2-siRNA^{NTC} with Bonferroni's multiple comparison correction (ns – not significant, **p<0.01).

C. Albumin-binding properties of siRNA conjugates in human CSF. Cy5-labeled siRNA, Chol-siRNA, or L2-siRNA (1 μM) was mixed with 300 μl of human CSF and analyzed using fast protein liquid chromatography (FPLC). Only L2-siRNA elutes in known albumin-containing fractions. Same N=1 CSF sample used for each condition.

D. L2-siRNA associates with albumin in CSF from patients with Alzheimer's Disease (AD) as well as those without neurodegeneration (N=3 control and AD samples). Error bars represent SD.

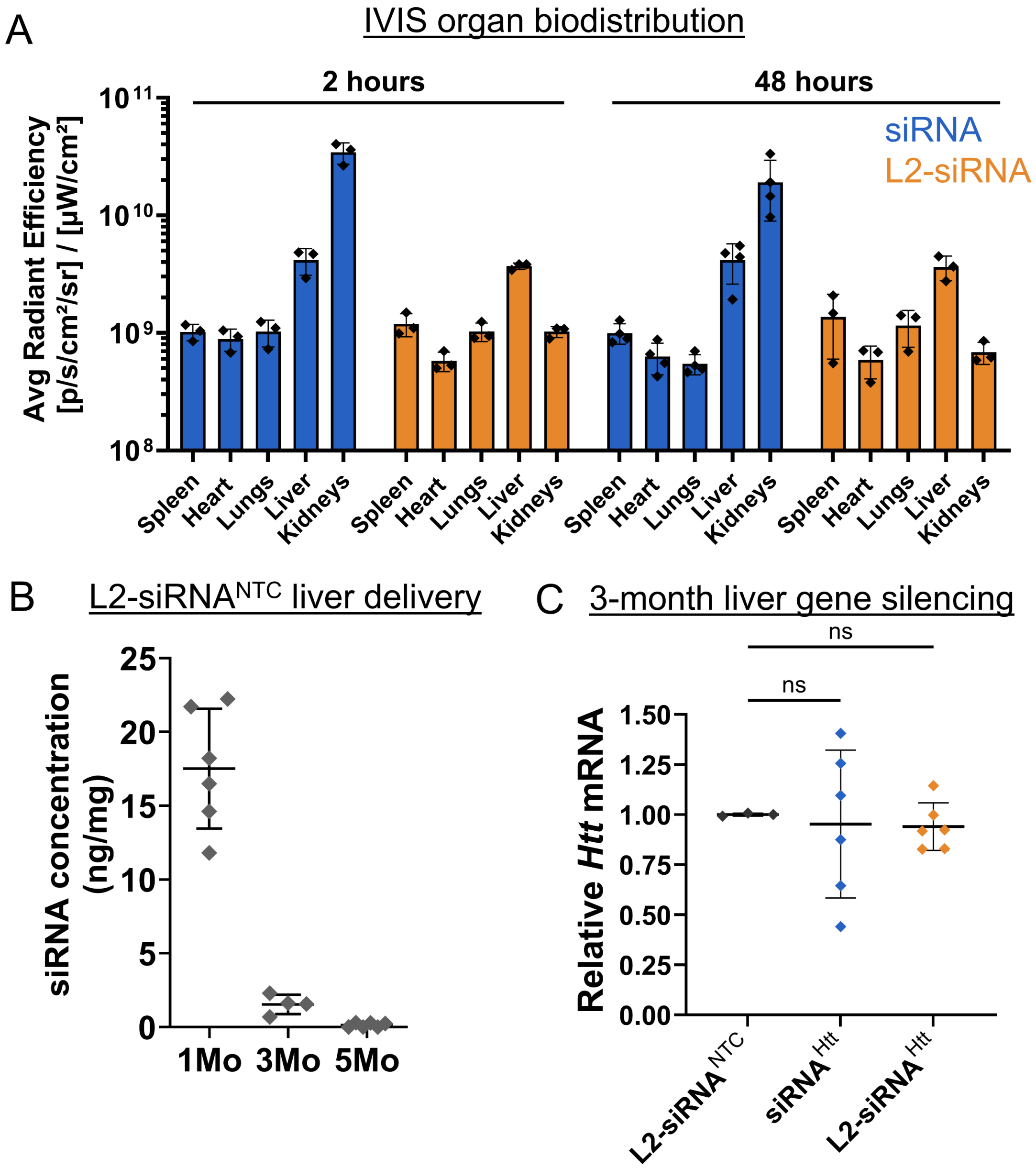

Supplementary Figure S2. Clearance to peripheral organs

A. Accumulation in peripheral organs assessed by In Vivo Imaging System (IVIS) 2 and 48 hours after ICV injection of Cy5-labeled siRNA or L2-siRNA (7.5-10 nmol). Average radiant efficiency of Cy5 fluorescence is reported. N=3-4 mice.

B. L2-siRNA^{NTC} liver delivery 1, 3, and 5 months (Mo) after ICV injection (15 nmol) measured with the PNA assay. Values below the limit of detection were plotted at 0. N=4-6 mice.

C. *Htt* mRNA expression levels in the liver as measured by RT-qPCR 3 months after ICV injection (15 nmol). Each point represents an individual mouse (N=3-6). Significance was calculated as a one-way ANOVA compared to L2-siRNA^{NTC} with Bonferroni's correction for multiple comparisons. Data presented as mean ± SD in every graph (ns - not significant).

48-hour ICV biodistribution through deep brain structures

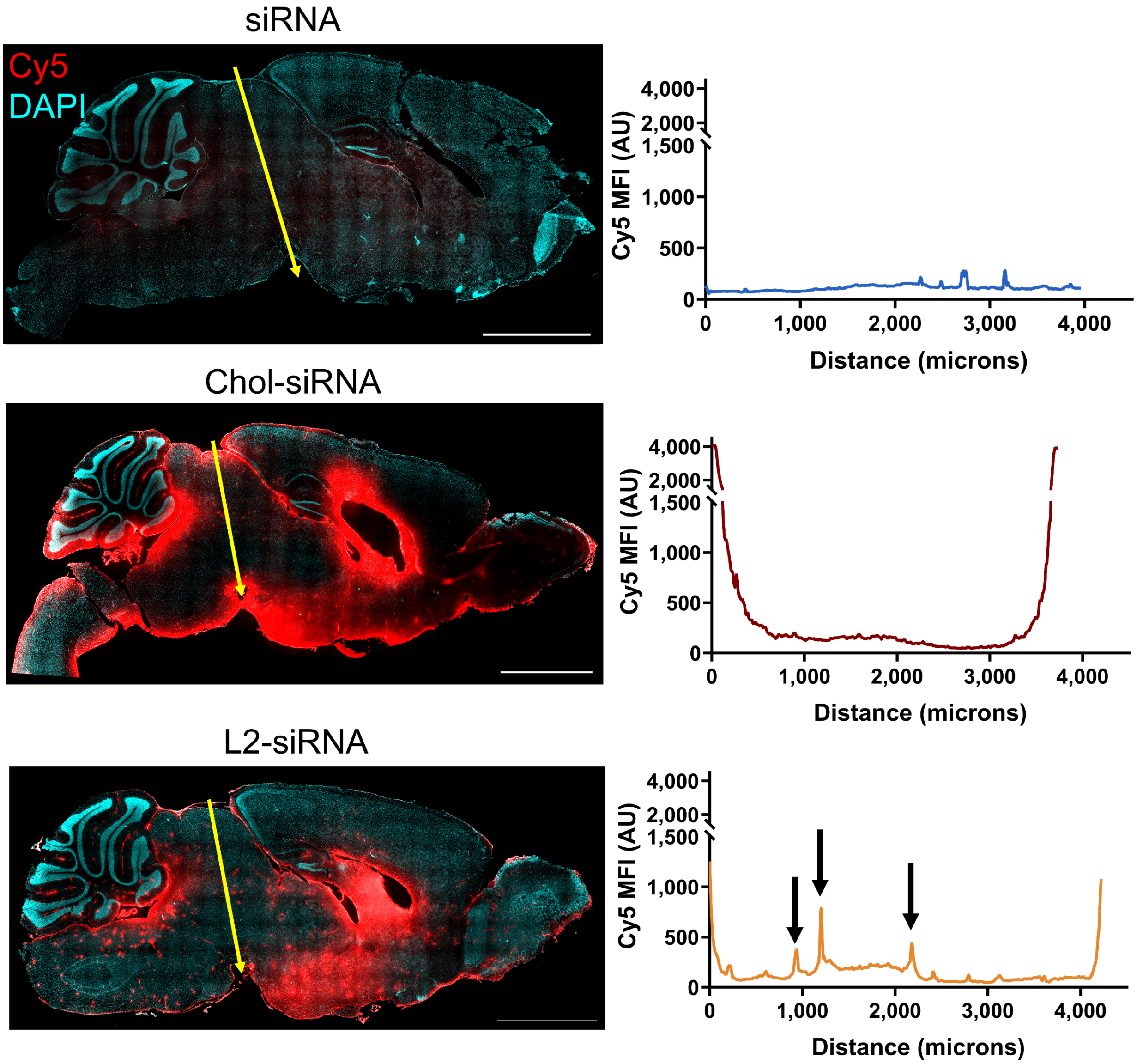

Supplementary Figure S3. Additional replicates of biodistribution after ICV delivery

Delivery throughout CNS 48 hours after ICV delivery. Cy5 mean fluorescence intensity (MFI) plotted along distance indicated by yellow line. Distribution shown for siRNA (A), Chol-siRNA (B), and L2-siRNA (C). Section thickness = 30 μm, scale bars = 2.5 mm. Arrows indicate deep brain structure regions predicted to be associated with perivascular transport.

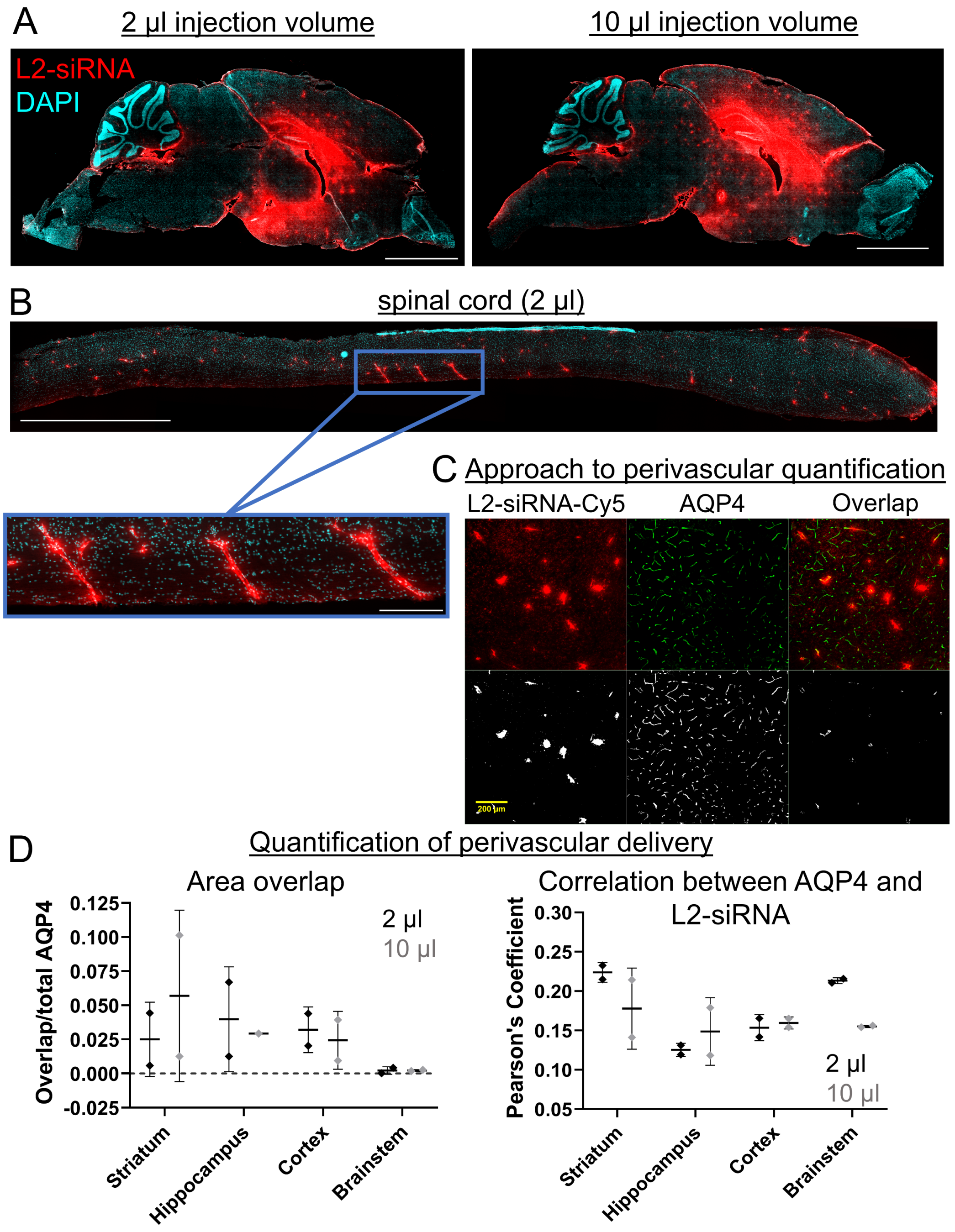

Supplementary Figure S4. L2-siRNA delivery to perivascular spaces is observed with low injection volumes

A-B. To test whether perivascular delivery is an artifact of injection volume, mice were administered an equivalent ICV dose of L2-siRNA (2 nmol), in either 2 µl or 10 µl by adjusting the initial compound concentration appropriately. After 48 hours, biodistribution was examined in the parenchyma (panel A) and spinal cord (panel B), with L2-siRNA shown in red, counterstained with DAPI in cyan (30 µm sections, scale bar = 2.5 mm).

C-D. Quantification of L2-siRNA delivery to perivascular spaces computed as the colocalization of AQP4, demarcating the outer boundary of the PVS, with Cy5-tagged L2-siRNA. Area overlap is determined by the Mander's coefficient, which reflects the fraction of AQP4 staining that is Cy5 positive. The overall association between the signals was determined using Pearson's correlation with predefined regions of interest for different brain regions. Scale bar = 200 μm. N=2 mice per treatment, mean ± SD.

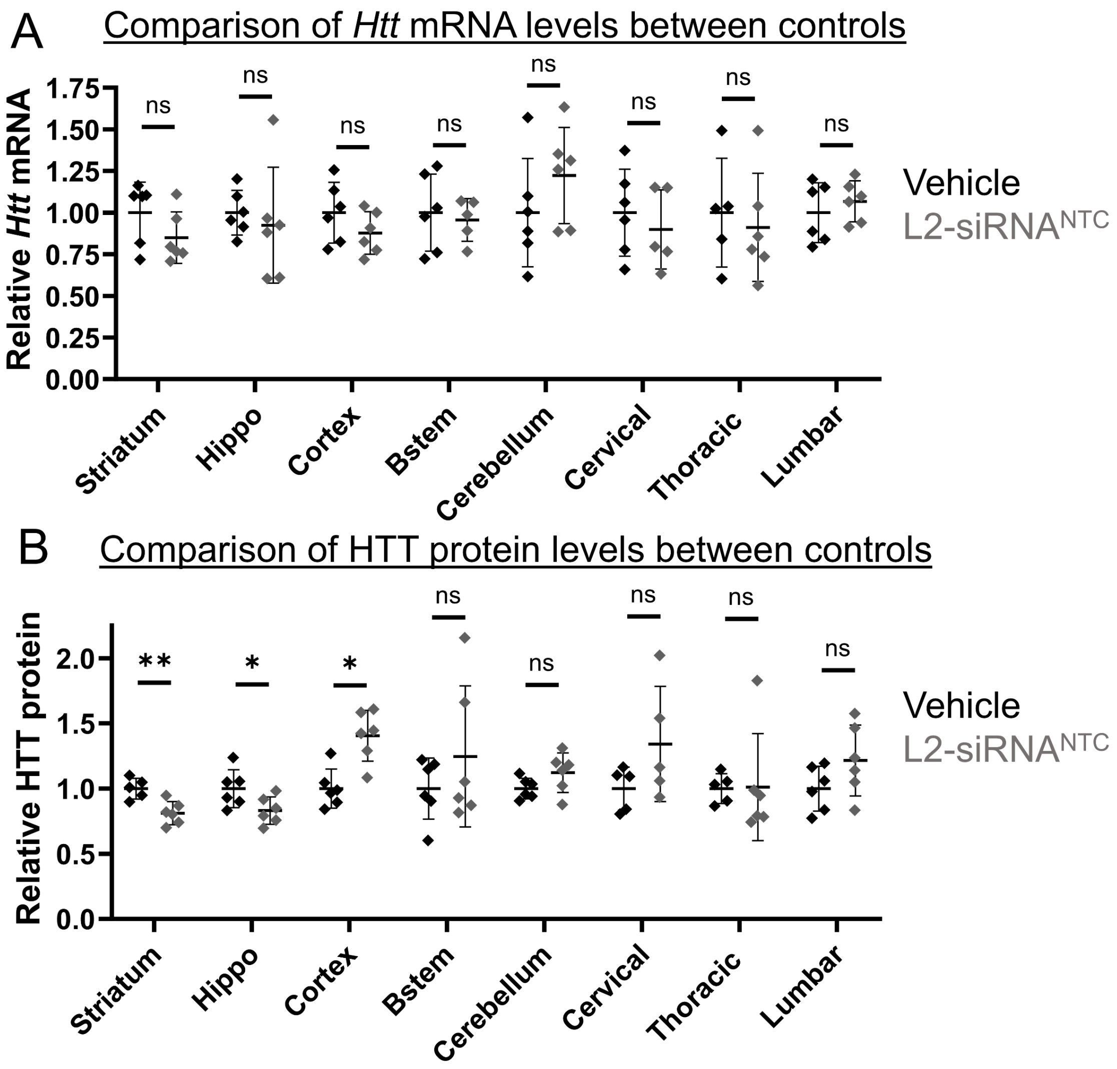

Supplementary Figure S5. Comparison of Htt expression between negative controls

Knockdown of Htt was assessed one month after ICV injection of vehicle (0.9% NaCl) or L2-siRNA^{NTC} (15 nmol) at the mRNA level by RT-qPCR (A) and protein level by western blot (B). Each data point represents one mouse (N=6), is normalized to vehicle, and represented as mean ± SD. Statistics were computed as unpaired, two-tailed t-tests (ns – not significant, *p<0.05, **p<0.01). Hippo = hippocampus, Bstem = brainstem.

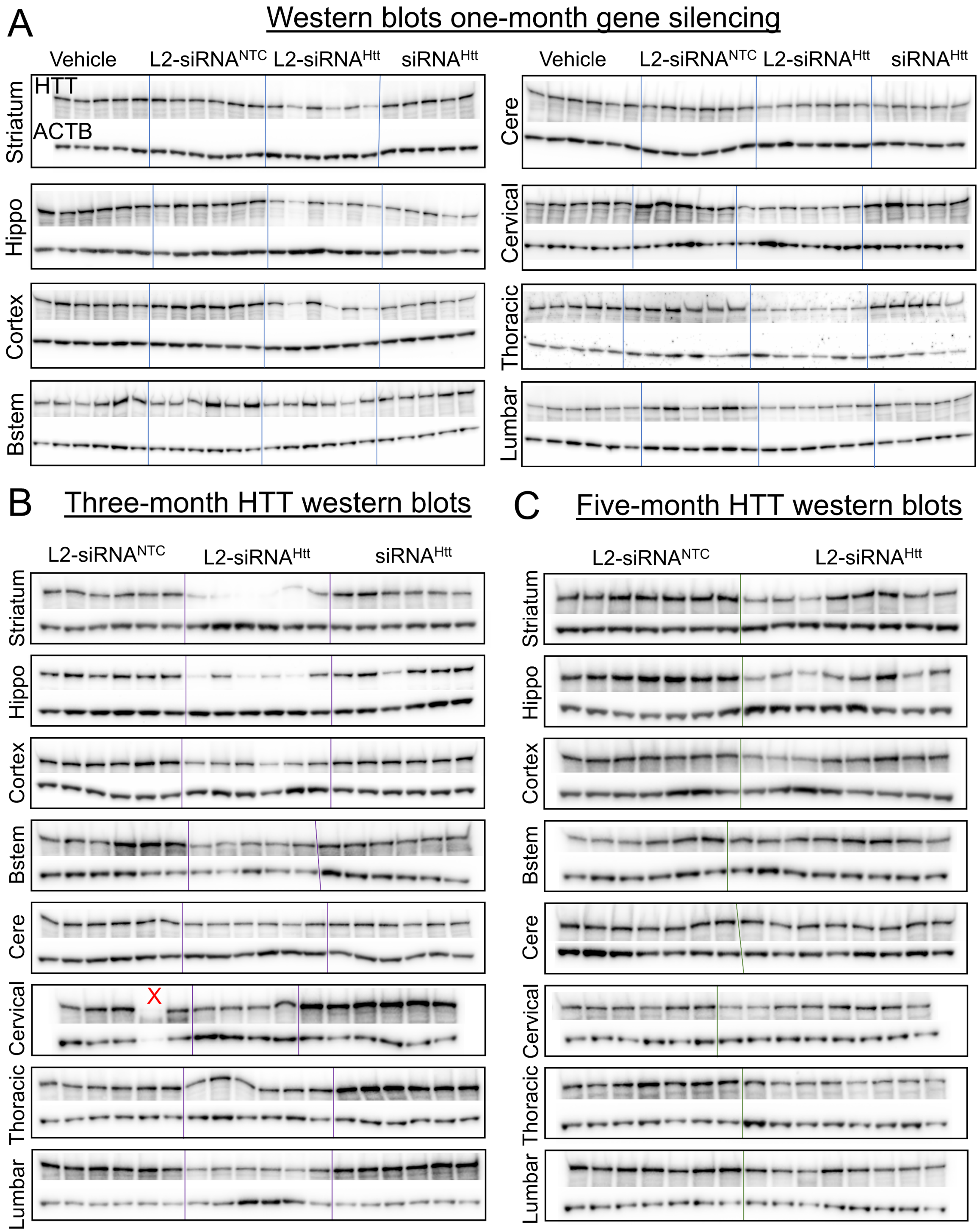

Supplementary Figure S6. Raw western blots to assess HTT protein silencing

Western blots associated with protein knockdown quantified in Figure 2C. For all boxes, the top bands are HTT and the bottom bands are the housekeeping protein beta-actin (ACTB). Each band represents an individual mouse (i.e. biological replicate). Blots are shown for each region and timepoint. Red X marks a sample with degraded protein that was excluded from analysis.

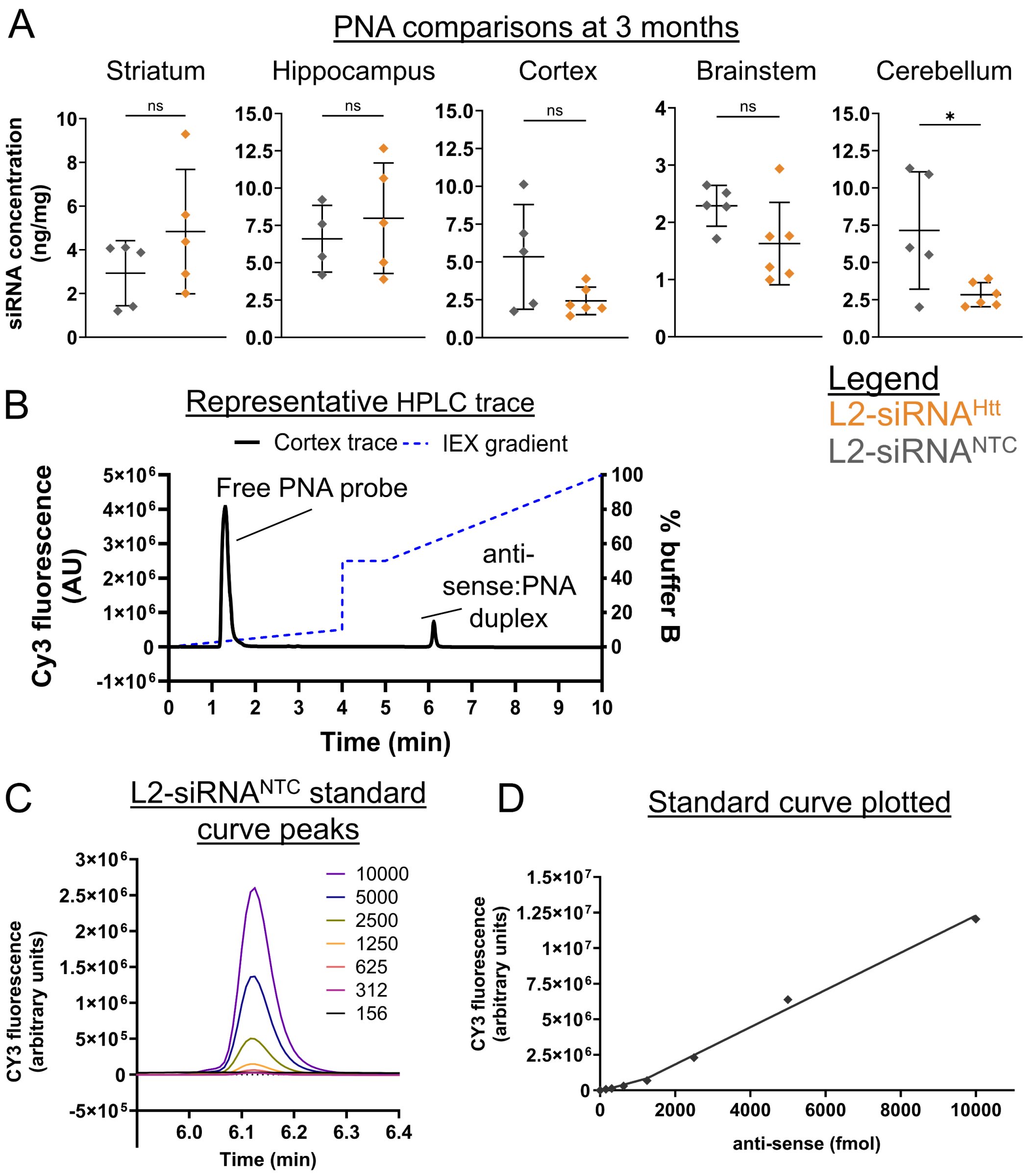

Supplementary Figure S7. PNA assay supporting information

A. Comparing siRNA delivery between L2-siRNA^{NTC} and L2-siRNA^{Htt} 3 months after ICV injection (15 nmol). Units are nanograms (ng) of anti-sense strand per milligram (mg) of tissue. Mean ± SD from N=5-6 biological replicates, two-tailed unpaired t-tests for each region (ns – not significant, *p<0.05).

B. Representative HPLC trace showing elution of anti-sense:PNA complex overlayed with ion-exchange gradient (dashed blue line). Trace shown from a cortex sample of L2-siRNA^{NTC}.

C. Raw peaks for L2-siRNA^{NTC} standard curve (in fmol units).

D. Quantification of peaks represented with a bilinear fit for L2-siRNA^{NTC} standard curve.

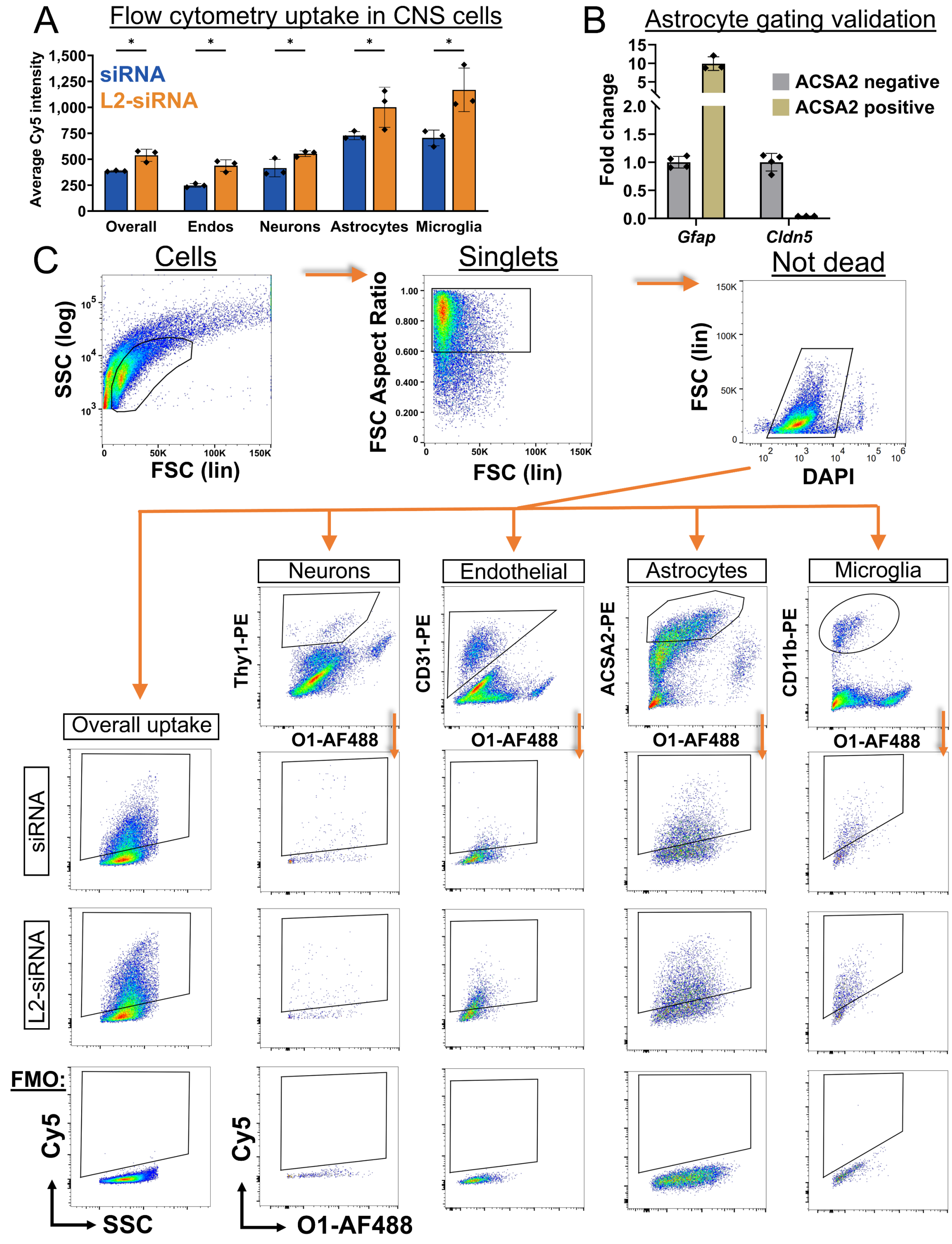

Supplementary Figure S8. Flow cytometry assessment of cell-specific uptake

A. Cell-specific uptake assessed 48 hours after ICV injection of L2-siRNA or siRNA (10 nmol). Data reported as mean Cy5 intensity of live cells. Statistics computed as unpaired, two-tailed

t-tests corrected for multiple comparisons using the false discovery rate (FDR) approach with Q=5%. N=3 mice, data presented as mean ± SD.

B. Validation of astrocyte gating strategy by performing RT-qPCR on fluorescence activated cell sorting (FACS) purified ACSA2+/O1- and ACSA2-/O1- cells. N=3-4 mice.

C. Gating strategy for identifying cell types and measuring Cy5 uptake. All samples are pre-gated to separate cells from debris, single cells from doublets, and live cells from DAPI+ dead cells. Neurons are defined as Thy1+/O1-, endothelial cells as CD31+/O1-, Astrocytes as ACSA2+/O1, Microglia/macrophages as CD11b+/O1-. Within these cell populations, Cy5+ events are gated using fluorescence-minus one (FMO) controls unique to each cell type. Representative plots are shown (median of each triplicate). AF488 = alexafluor 488, PE = phycoerythrin, FSC = forward scatter, SSC = side scatter.

A Quality control and filtering of CD11b^{pos} cells

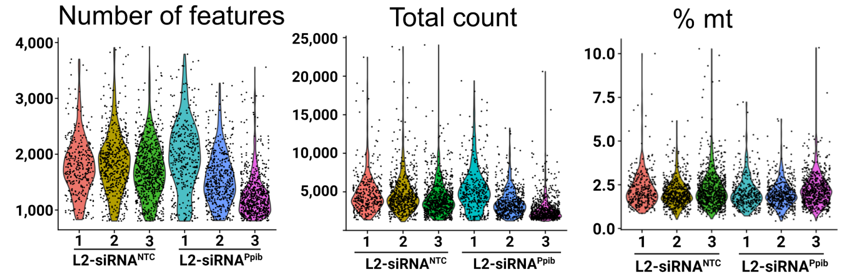

B Cell-type annotation

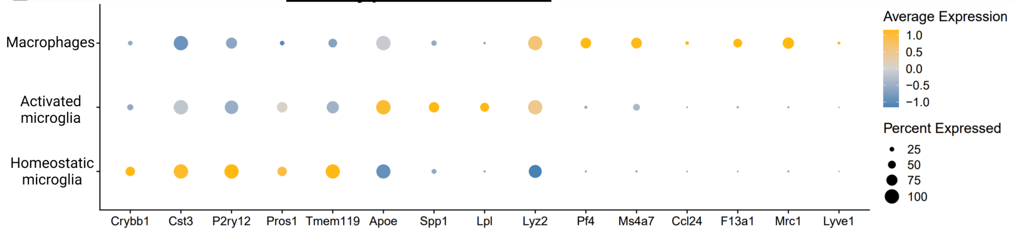

C Table of cell yield per sample

	Total CD11b+	Homeostatic microglia	Activated microglia	Macrophages	Unknown
L2-siRNA^{NTC} 1	343	293	28	18	4
L2-siRNA^{NTC} 2	573	480	53	29	11
L2-siRNA^{NTC} 3	771	645	72	49	5
L2-siRNA^{Ppib} 1	368	319	25	23	1
L2-siRNA^{Ppib} 2	505	437	24	33	11
L2-siRNA^{Ppib} 3	701	615	25	51	10

D *Ppib* expression density plots

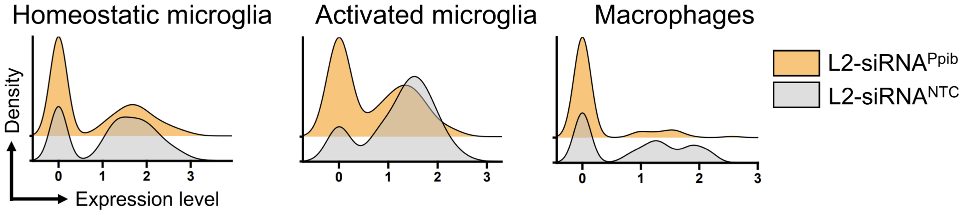

Supplementary Figure S9. CD11b^{pos} scRNA-seq supporting information

A. Standard quality control metrics were employed for each sample processed with T2 PIPseq kits. Cells were filtered based on number of features, keeping those containing 800-4,000 uniquely expressed genes. Total count represents the absolute number of transcripts detected and percent mitochondrial (% mt) reads is the fraction of transcripts associated with mitochondrial mRNA. Created with BioRender (https://BioRender.com/i7ldgb4).

B. Dot plot of canonical Cd11b^{pos} population markers.

C. Number of cells identified in each CD11b^{pos} population for all replicates.

D. Smoothed ridgeline density plots showing changes in *Ppib* expression between L2-siRNA^{NTC} and L2-siRNA^{Ppib}.

A Quality control and filtering of CD11b^{neg}

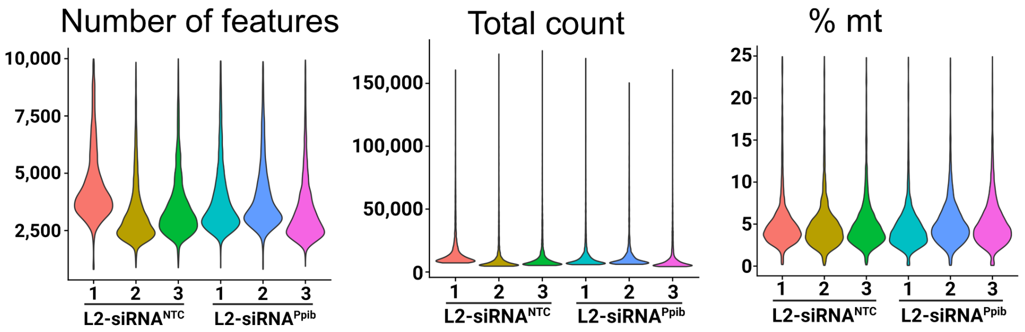

B Table of cell yield per sample

	L2-siRNA^{NTC}	L2-siRNA^{Ppib}		L2-siRNA^{NTC}	L2-siRNA^{Ppib}
Smooth muscle cells	221	300	Dural border cells	508	368
Endo/Pericytes	284	231	Inner arachnoid cells	107	108
Endo (arterial)	446	517	Arachnoid barrier cells	33	35
Endo (venous/capillary)	1015	1310	Parenchymal perivascular fibroblasts	64	77
ChP epithelial cells	2481	2259	Pial fibroblasts	50	48
Ependymal cells	2725	2195	Olfactory ensheathing cells	101	353
Oligodendrocytes	2548	2368	Neural IPCs	359	308
Astrocytes	770	1965	Neurons	182	104
Bergmann glia	960	416	Microglia	62	50

C Ridgeline plots of *Ppib* expression

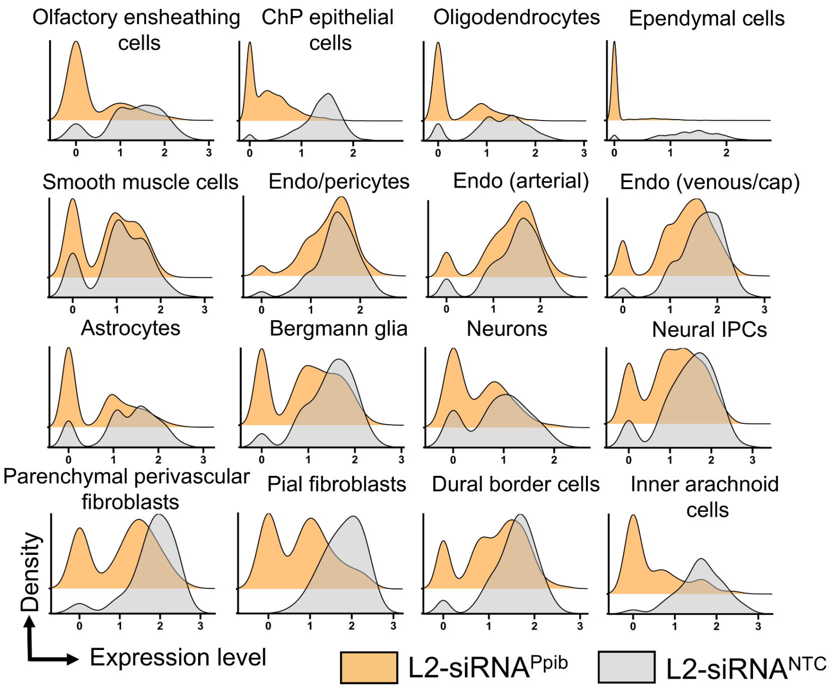

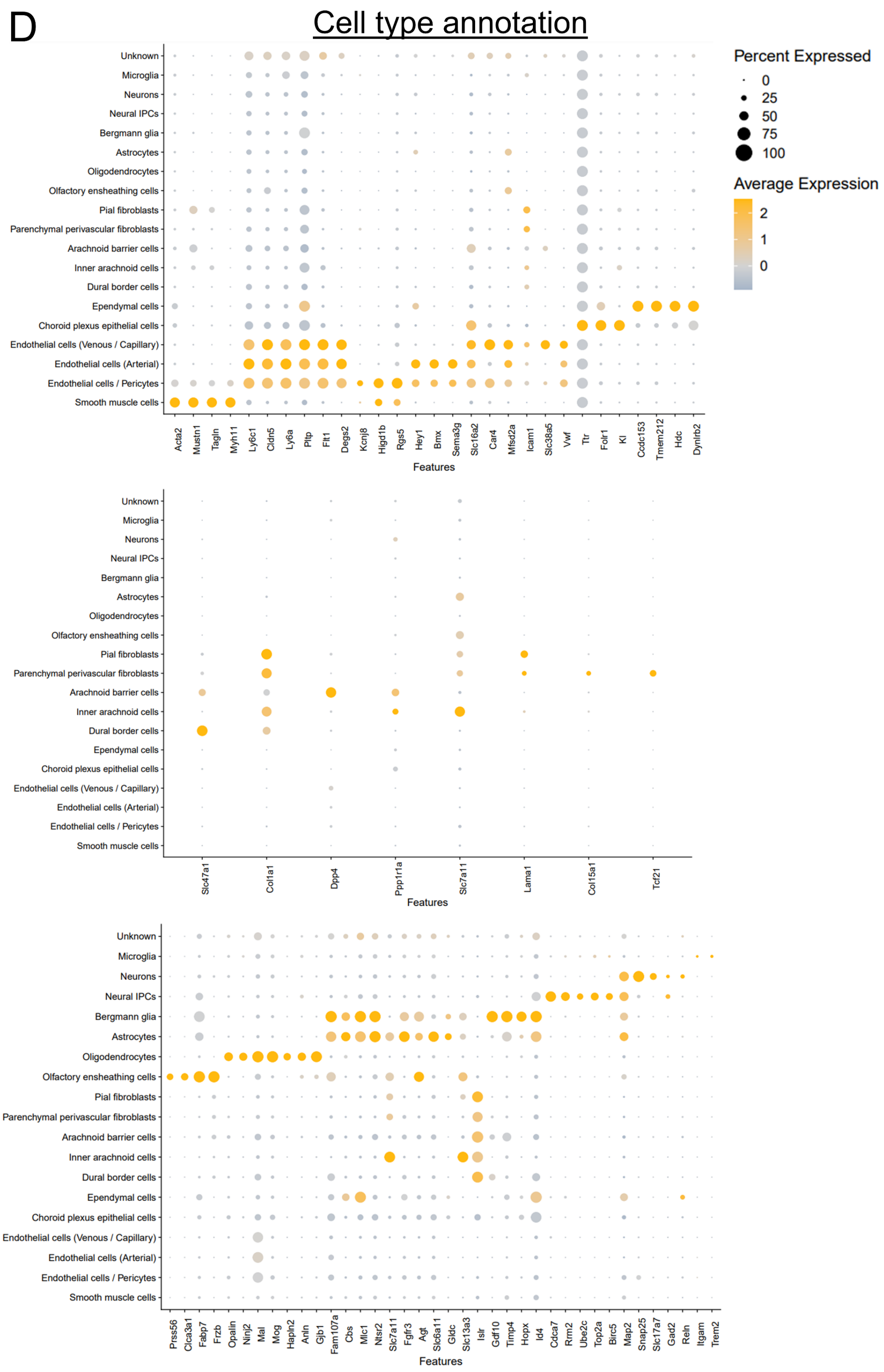

D
Cell type annotation
Percent Expressed
0
25
50
75
100
Average Expression
2
1
0
Unknown
Microglia
Neurons
Neural IPCs
Bergmann glia
Astrocytes
Oligodendrocytes
Olfactory ensheathing cells
Pial fibroblasts
Parenchymal perivascular fibroblasts
Arachnoid barrier cells
Inner arachnoid cells
Dural border cells
Ependymal cells
Choroid plexus epithelial cells
Endothelial cells (Venous / Capillary)
Endothelial cells (Arterial)
Endothelial cells / Pericytes
Smooth muscle cells
Acta2 Mustn1 Tagln Myh11 Ly6c1 Cldn5 Ly6a Pltp Flt1 Degs2 Kcnj8 Higd1b Rgs5 Hey1 Bmx Sema3g Slc16a2 Car4 Mfsd2a Icam1 Slc38a5 Vwf Ttr Folr1 Kl Ccdc153 Tmem212 Hdc Dynlrb2
Features
Slc47a1 Col1a1 Dpp4 Ppp1r1a Slc7a11 Lama1 Col15a1 Tcf21
Features
Prss56 Clca3a1 Fabp7 Frzb Opalin Ninj2 Mal Mog Hapln2 Anln Gjb1 Fam107a Cbs Mlc1 Ntsr2 Slc7a11 Fgfr3 Agt Slc6a11 Gldc Slc13a3 Islr Gdf10 Timp4 Hopx Id4 Cdca7 Rrm2 Ube2c Top2a Birc5 Map2 Snap25 Slc17a7 Gad2 Reln Itgam Trem2
Features

Supplementary Figure S10. CD11b^{neg} scRNA-seq supporting information

A. Standard quality control metrics were examined for each sample processed with T20 PIPseq kits. Cells were filtered based on number of features, keeping those containing 800-10,000 uniquely expressed genes. Created in BioRender (https://BioRender.com/nfbiele).

B. Table of cell yield for L2-siRNA^{NTC} and L2-siRNA^{Ppib}, reflecting the sum of cells from N=3 samples for each treatment.

C. Smoothed ridgeline density plots of *Ppib* expression for L2-siRNA^{NTC} and L2-siRNA^{Ppib} in each CD11b^{neg} population identified.

D. Dot plot of canonical Cd11b^{neg} population markers.

A Macrophage subclusters

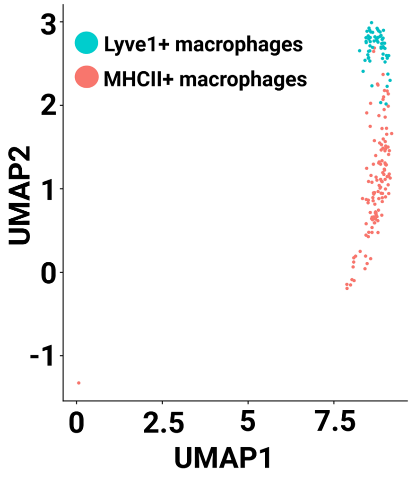

B Cell yield per sample

	MHCII+	Lyve1+
L2-siRNA^{NTC} 1	9	6
L2-siRNA^{NTC} 2	16	11
L2-siRNA^{NTC} 3	36	13
L2-siRNA^{Ppib} 1	13	8
L2-siRNA^{Ppib} 2	18	9
L2-siRNA^{Ppib} 3	31	15

C Annotation of macrophage populations

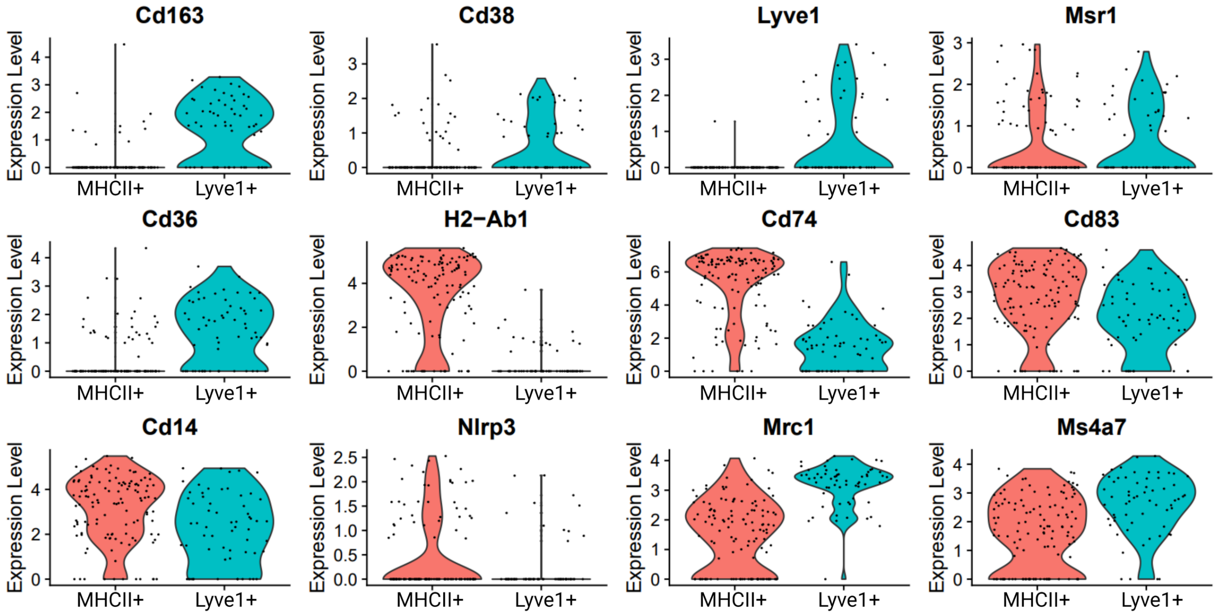

Supplementary Figure S11. Identifying macrophage subtypes

A. UMAP projection plot showing re-clustering of macrophages into Lyve1+ and MHCII+. Created in BioRender (https://BioRender.com/hvqjzib).

B. Number of cells identified in each macrophage population for all replicates combined.

C. Annotation of gene expression in Lyve1+ and MHCII+ macrophages. Created with Biorender (https://BioRender.com/qlpbu8b)

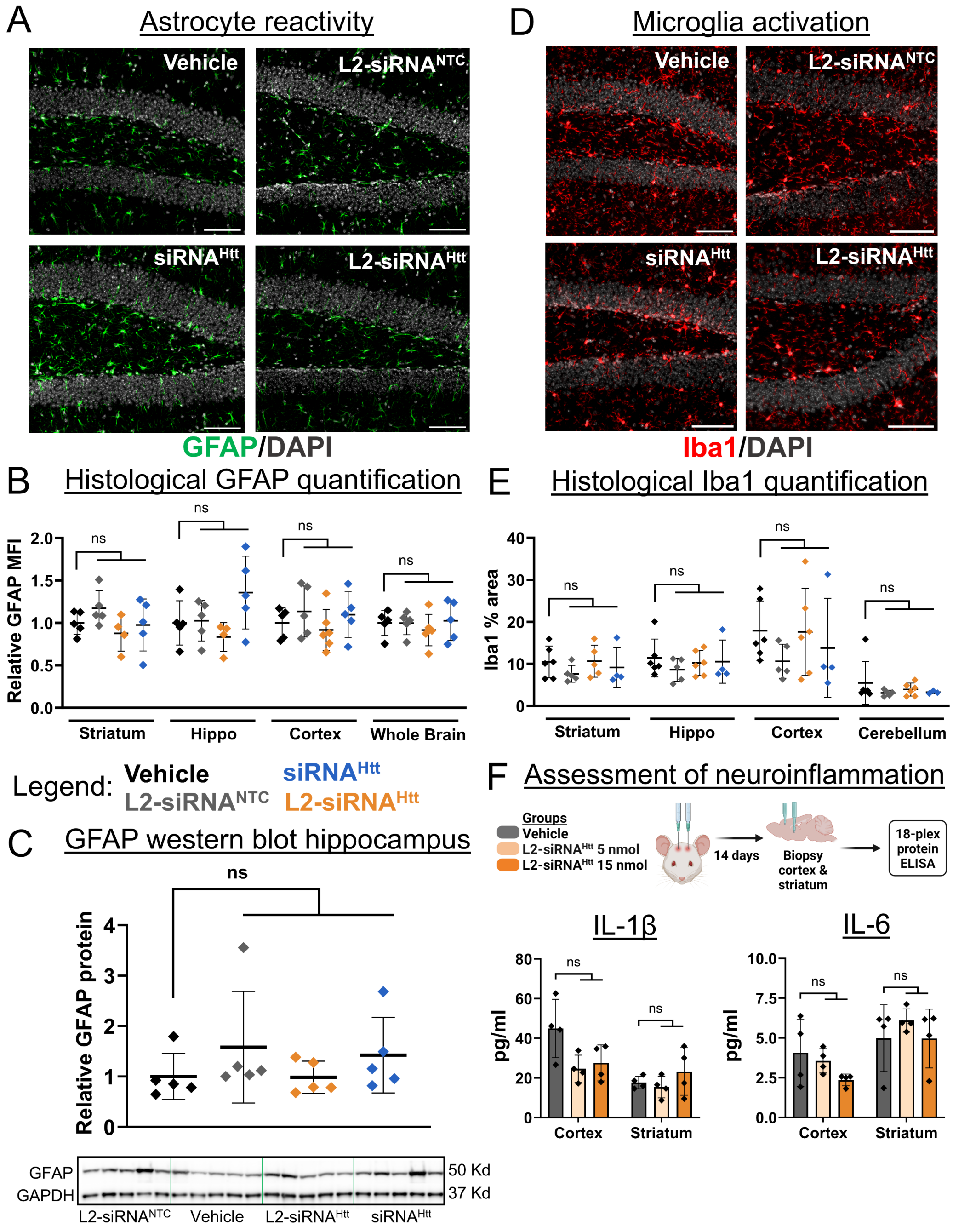

Supplementary Figure S12. L2-siRNA does not promote CNS toxicity

A. Mice administered 15 nmol of compounds (or 0.9% saline) and stained for GFAP 1 month after ICV injection. Representative images of the hippocampal dentate gyrus are shown. Section thickness = 8 µm, scale bar = 100 µm.

B. Quantification of GFAP mean fluorescence intensity (MFI) normalized to vehicle across different brain regions. Data are presented as mean ± SD, with N=4-5 mice per condition. One-way ANOVA with Bonferroni's correction was performed for each region.

C. GFAP levels measured by Western blot, normalized to GADPH housekeeping gene, and then normalized to vehicle control. Raw western blot displayed for GFAP and GAPDH. N=5 mice statistically analyzed with a one-way ANOVA and Bonferroni's correction.

D. Mice were administered 15 nmol of compounds (or 0.9% saline) and stained for Iba1 1 month after ICV injection. Representative images of the hippocampal dentate gyrus are shown. Section thickness = 8 μm, scale bar = 100 μm.

E. Quantification of Iba1 protein levels normalized to vehicle. N=4-6 mice per condition. One-way ANOVA with Bonferroni's correction was performed for each region compared to vehicle.

F. Levels of inflammatory cytokines assessed two weeks after ICV injection of vehicle (0.9% saline) or L2-siRNA^{Htt} (5 nmol or 15 nmol). Additional cytokine levels are shown in Supplementary Figure S13. N=4 mice per condition. One-way ANOVA with Bonferroni's correction was performed for each region and cytokine (ns – not significant). All data are presented as mean ± SD. Created with BioRender (https://BioRender.com/4wiuuuy).

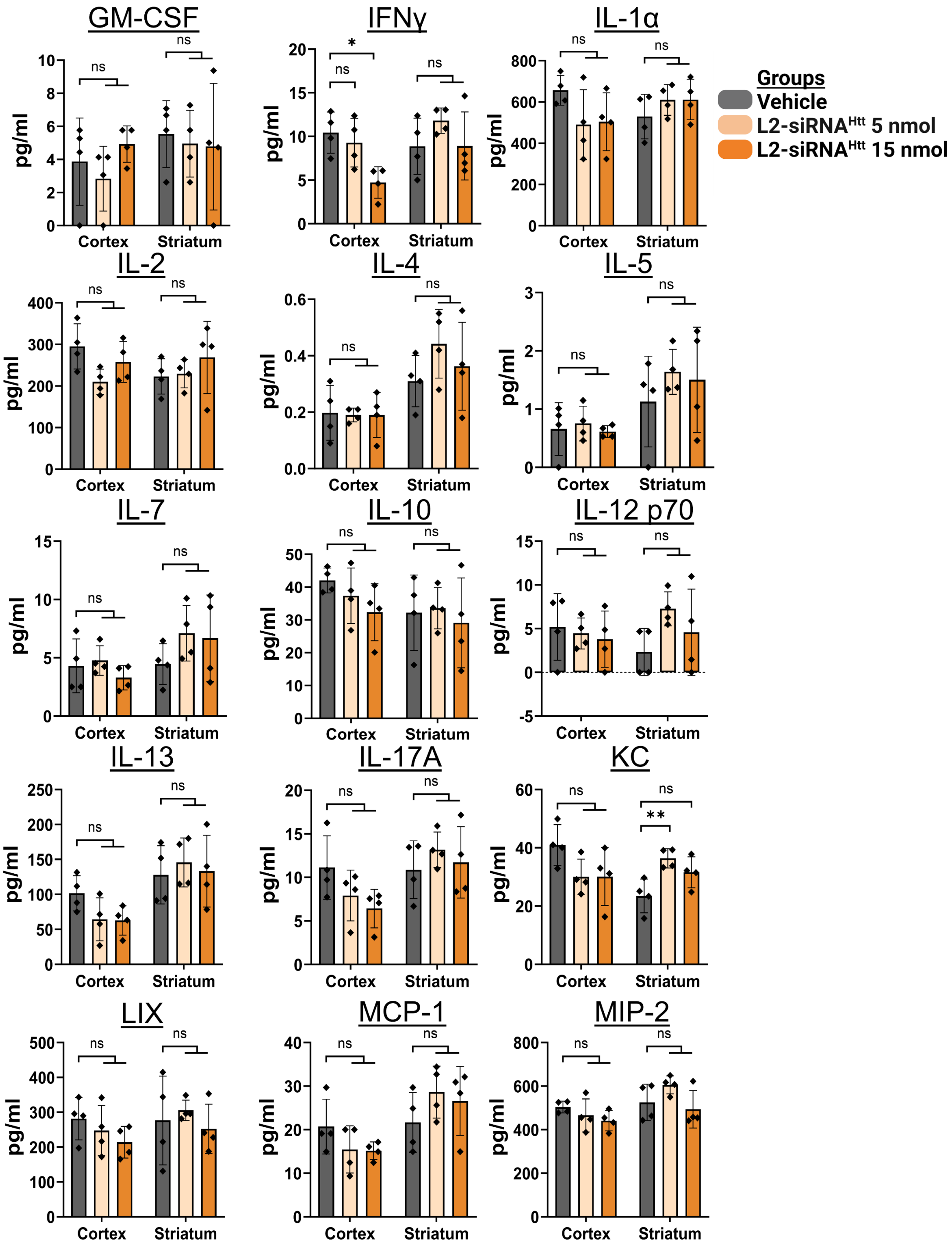

Supplementary Figure S13. Inflammatory protein ELISA panel

Two weeks after ICV injection of vehicle (0.9% NaCl) or L2-siRNA^{Htt} (5 nmol or 15 nmol) in mice, cortex and striatum homogenates were analyzed for presence of inflammatory proteins (cytokines, chemokines, growth factors). All samples below the limit of detection on the standard curve were treated as 0 in terms of graphical presentation and statistical analysis. All samples were below the limit of detection for TNFα, and therefore data are not shown. All data are presented as mean ± SD. A one-way ANOVA with Bonferroni's correction was performed for each

region and protein (ns – not significant, *p<0.05, **p<0.01). GM-CSF, granulocyte-macrophage colony stimulating factor; IFNγ, interferon gamma; KC, keratinocyte chemoattractant; LIX, Lipopolysaccharide-induced CXC chemokine; MCP-1, monocyte chemoattractant protein-1; MIP-2, macrophage inflammatory protein-2.

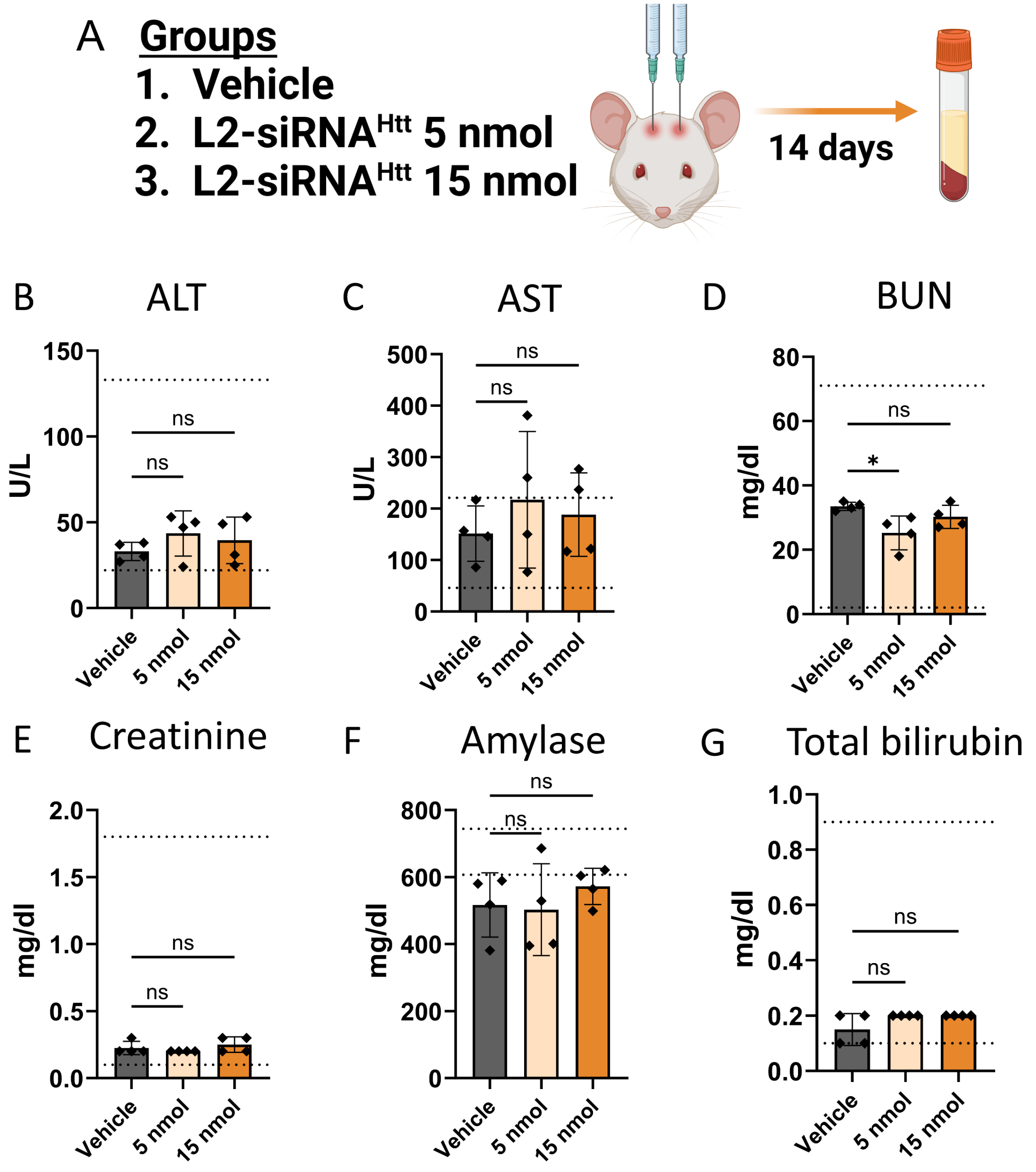

Supplementary Figure S14. Serum chemistry assessment of organ toxicity in mice

Mice were injected ICV with a vehicle (0.9% NaCl) or L2-siRNA^{Htt} (5 nmol or 15 nmol), and blood serum was isolated after two weeks. Six standard markers were measured in this panel: alanine aminotransferase (ALT), aspartate aminotransferase (AST), blood urea nitrogen (BUN), creatinine, amylase, and total bilirubin. Data are presented as mean ± SD. Statistics are computed as a one-way ANOVA with Bonferroni's correction compared to vehicle (ns – not significant, *$p<0.05$). Standard chemistry reference range was obtained from UCLA Division of Laboratory

Animal Medicine and is represented by dotted lines. Panel A created in BioRender (https://biorender.com/afpd912).

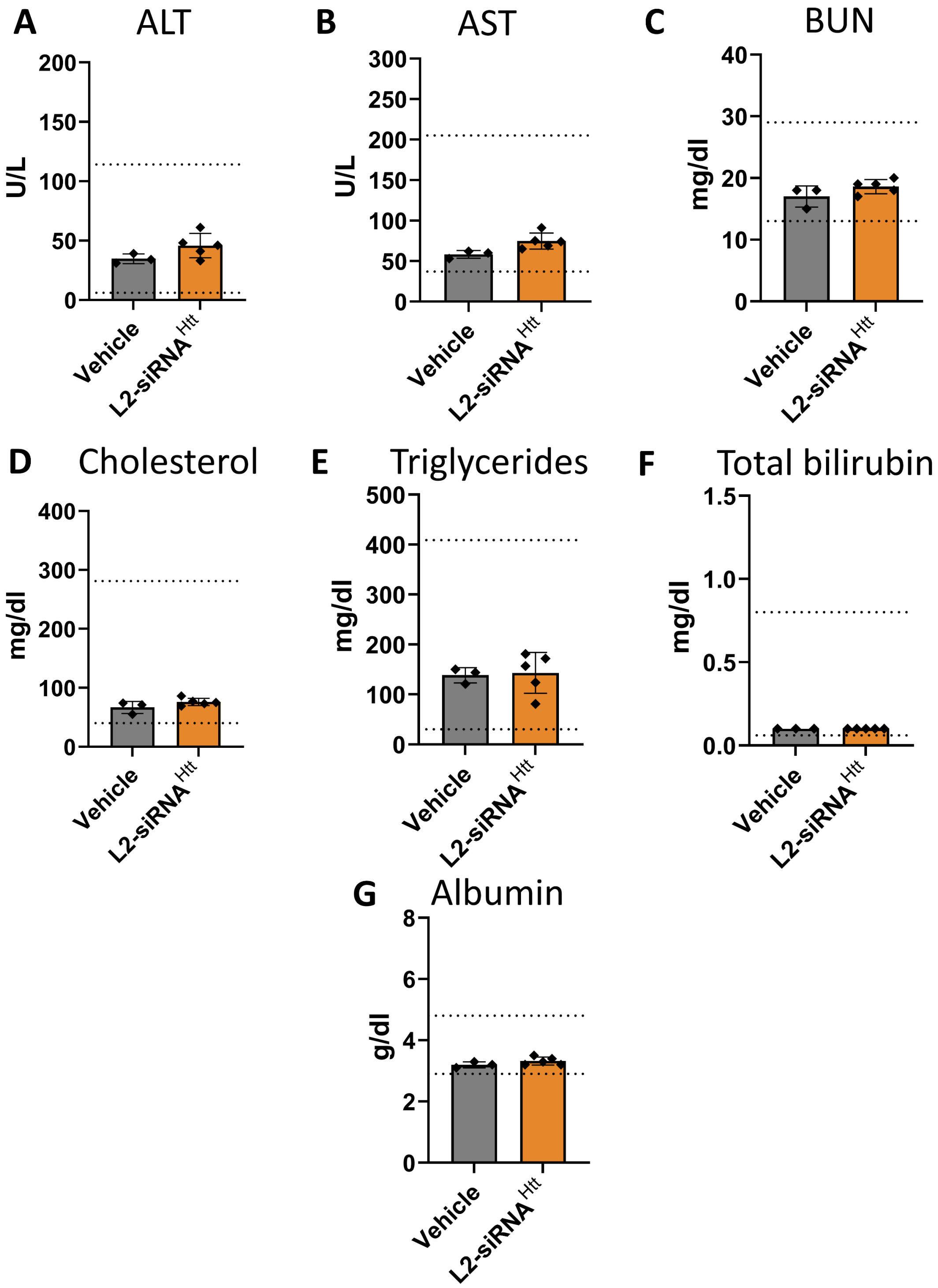

Supplementary Figure S15. Serum chemistry assessment of organ toxicity in rats

Rats were injected intrathecal with 60 nmol L2-siRNA^{Htt} or vehicle (0.9% NaCl) and serum was collected after one-month. Abbreviations: alanine aminotransferase (ALT), aspartate aminotransferase (AST), blood urea nitrogen (BUN). Reference range for Sprague Dawley rats was obtained from UCLA Division of Laboratory Animal Medicine.

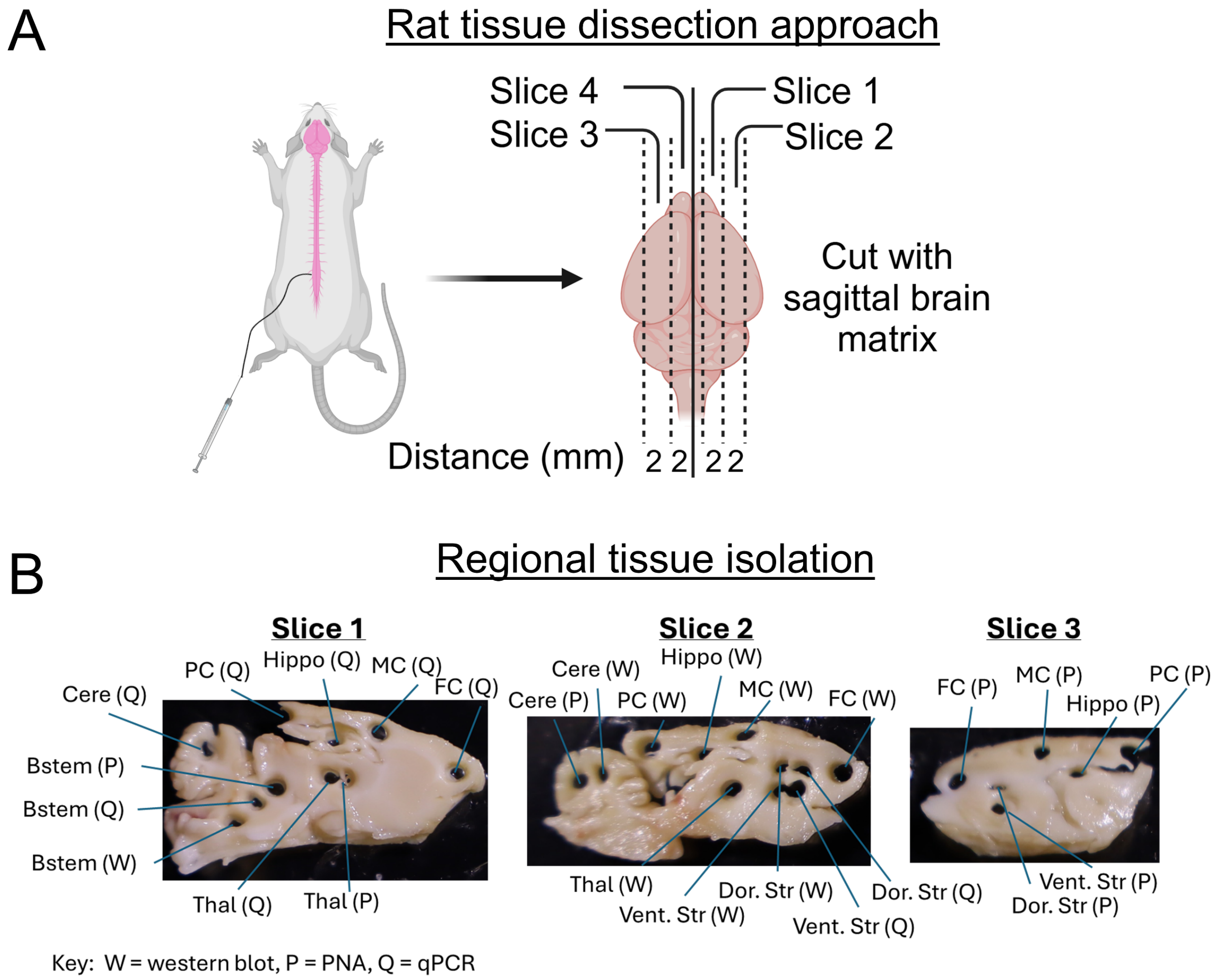

Supplementary Figure S16. Microdissection of rat brain regions

A. Due to challenges associated with minute variation in microdissection, we standardized an approach to isolate each region of interest. The rat brains were sliced in a sagittal brain matrix (WBI RBMS-300S) pushing down one blade at a time from outside in. Gentle slicing back and forth of the blade prior to pushing down helped cut through the meninges and prevented folding-in of the cortex. Slice 4 was processed separately for histology. Slices 1-3 could be stored at -20°C in RNA later until further dissection. Created in BioRender (://BioRender.com/tqega7j).

B. First, the olfactory bulb is removed with a scalpel and split three ways for RT-qPCR, PNA, and WB. The cerebellum is biopsy-punched in the posterior lobe, avoiding the deep cerebellar nuclei. The cortical regions (posterior, medial, and frontal) were punched in between the white matter and meninges. The medial cortex was taken above the lateral ventricle. To isolate the hippocampus, it was peeled away from the meninges before biopsy-punching the center. The striatum was biopsy-punched from the second slice at the dorsal (below corpus collosum) and ventral (above anterior commissure) locations. All three biopsy punches for the brainstem were taken from slice #1. Lastly, the thalamus was located ventral to the hippocampus. The biopsy punches were stored in RNA later at -20°C until further processing.

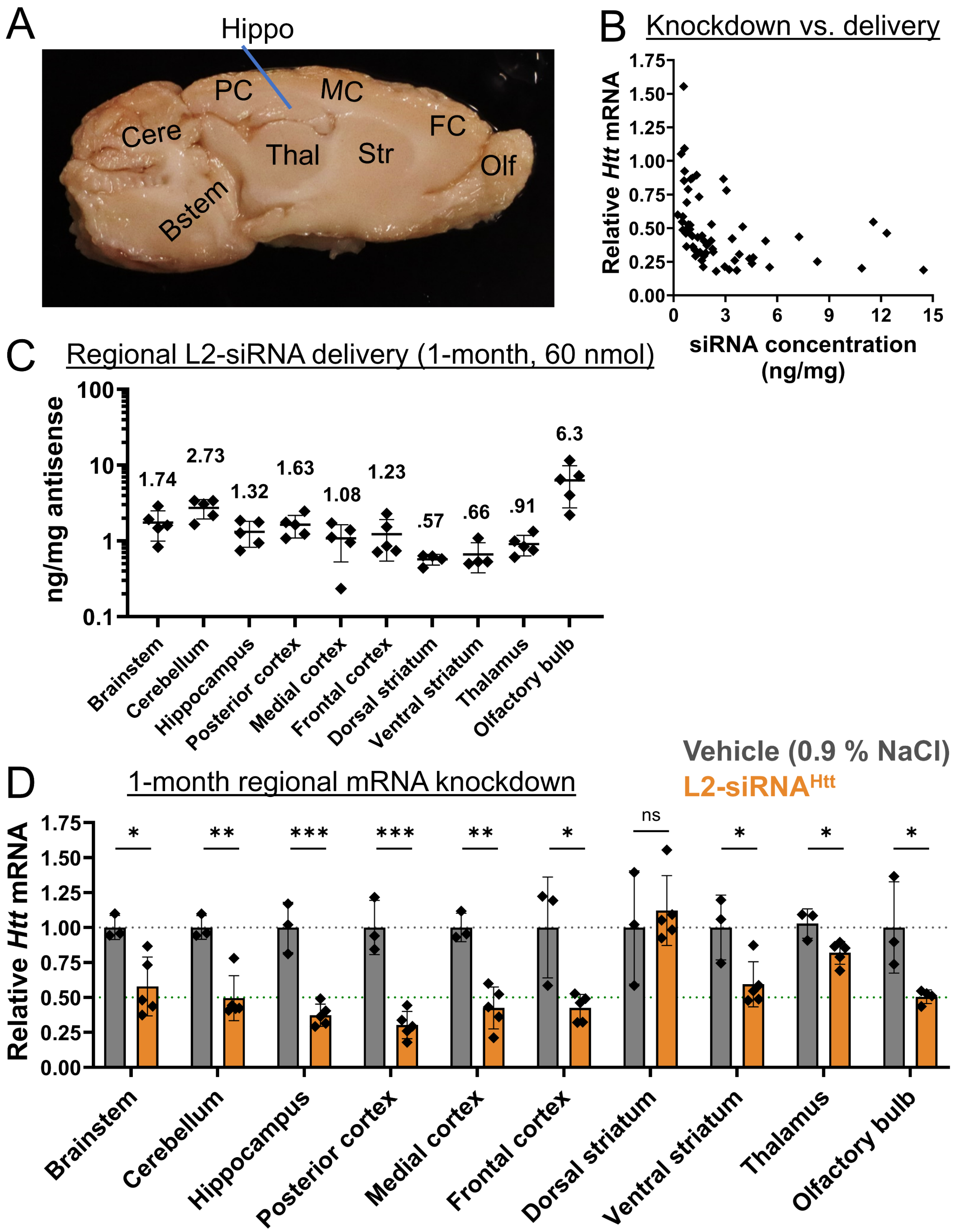
A
Hippo
PC
MC
Cere
Thal
Str
FC
Olf
Bstem
B Knockdown vs. delivery
Relative Htt mRNA
siRNA concentration (ng/mg)
C Regional L2-siRNA delivery (1-month, 60 nmol)
ng/mg antisense
1.74
2.73
1.32
1.63
1.08
1.23
.57
.66
.91
6.3
Brainstem
Cerebellum
Hippocampus
Posterior cortex
Medial cortex
Frontal cortex
Dorsal striatum
Ventral striatum
Thalamus
Olfactory bulb
D 1-month regional mRNA knockdown
Vehicle (0.9 % NaCl)
L2-siRNAHtt
Relative Htt mRNA
*
**
***
***
**
*
ns
*
*
*
Brainstem
Cerebellum
Hippocampus
Posterior cortex
Medial cortex
Frontal cortex
Dorsal striatum
Ventral striatum
Thalamus
Olfactory bulb

Supplementary Figure S17. Regional L2-siRNA delivery and huntingtin knockdown 1-month after intrathecal injection

A. Anatomical locations of brain regions on a representative rat brain slice. Bstem, brainstem; Cere, cerebellum; PC, posterior cortex; MC, medial cortex; FC, frontal cortex; Str, striatum; Hippo, hippocampus; Thal, thalamus; Olf, olfactory bulb.

B. Relationship between delivery and mRNA knockdown 1-month after intrathecal administration of L2-siRNA^{Htt} normalized to vehicle (800 µg ~ 60 nmol).

C. Absolute amount of antisense strand (ng) per milligram of brain tissue measured by the PNA assay 1 month after intrathecal injection of L2-siRNA^{Htt}. Each point represents a brain sample (N=5 rats). The mean is represented by a bar ± SD and is reported numerically for each region.

D. Regional *Htt* knockdown assessed 1-month after intrathecal injection of L2-siRNA^{Htt} (800 µg ~ 60 nmol). Each region is normalized to a vehicle control and statistics were computed as unpaired two-tailed t-tests for each region. Each point represents an individual biological replicate (i.e., a single rat) and data are presented as mean ± SD, N=3-5. (* $p<0.05$, ** $p<0.01$, *** $p<0.001$, ns – not significant)

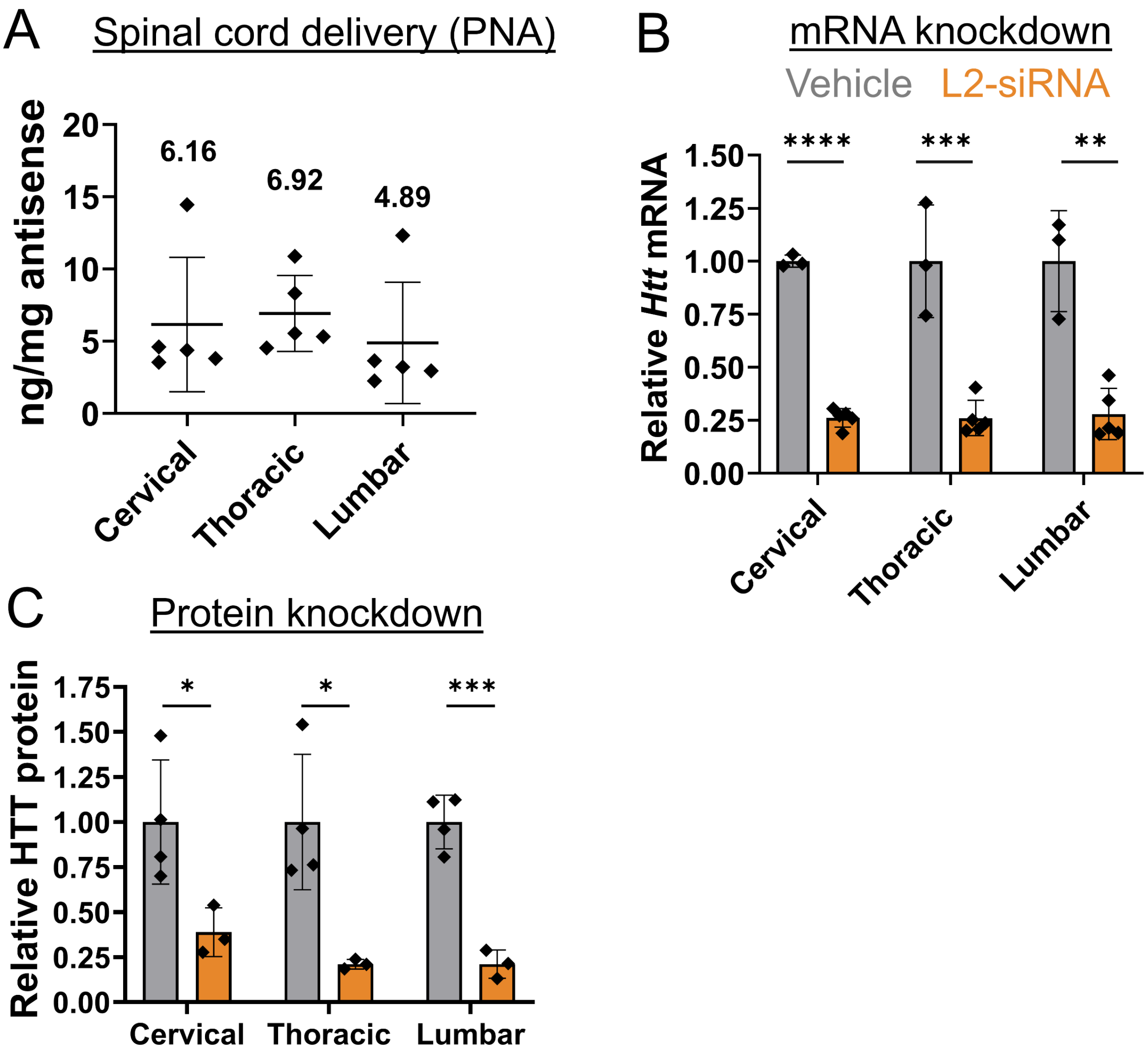

Supplementary Figure S18. L2-siRNA delivery and knockdown in spinal cord after intrathecal injection in rats

A. Delivery throughout the spinal cord was measured by PNA 1-month after intrathecal injection of L2-siRNA^{Htt} (800 μg ~ 60 nmol) (N=5 rats). Mean accumulation is reported for each region.

B. Regional *Htt* knockdown assessed by RT-qPCR.

C. HTT protein levels in the spinal cord as measured by western blot. Raw western blots are shown in Supplementary Figure S19. For panels B and C, statistical significance is calculated with unpaired two-tailed t-tests for each region compared to vehicle control. Each point represents an individual biological replicate (i.e., a single rat) and data are presented as mean ± SD, N=3-5. (* p<0.05, ** p<0.01, *** p<0.001, **** p<0.0001).

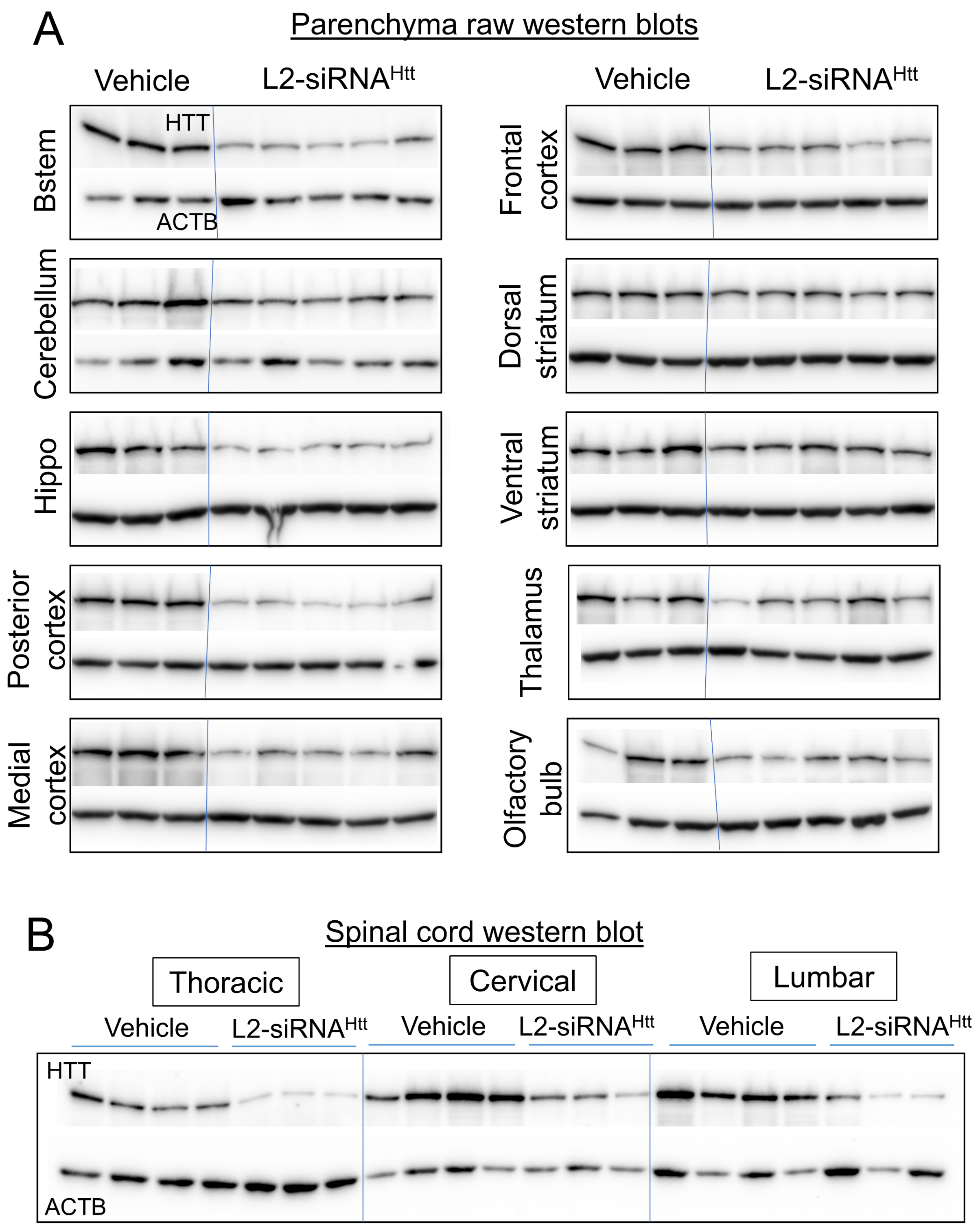

Supplementary Figure S19. Raw western blots from rat CNS

Western blots associated with protein knockdown quantified in Figure 6D and Supplementary Figure S18. For all boxes, the top bands are HTT and the bottom bands are the housekeeping protein beta-actin (ACTB). All blots are from a 1-month timepoint. Each band represents an individual rat.

Name	Sequence (5’->3’)	Location	Length	Ref
Htt S	(MeU)*(fA)*(MeU)(fA)(MeU)(fC)(MeA)(fG)(MeU)(fA)(MeA)(fA)(MeG)(fA)(MeG)(fA)(MeU)(fU)*(MeA)*(fA)	10150	20	Alterman et al. 2015
Htt AS	VP(meU)*(fU)*(MeA)(fA)(MeU)(fC)(MeU)(fC)(MeU)(fU)(MeU)(fA)(MeC)(fU)(MeG)(fA)(MeU)(fA)*(MeU)*(fA)	10150	20	Alterman et al. 2015
PPIB S	(MeA)*(fA)*(MeC)(fA)(MeG)(fC)(MeA)(fA)(MeA)(fU)(MeU)(fC)(MeC)(fA)(MeU)(fC)(MeG)(fU)*(MeG)*(fA)	437	20	Reynolds et al. 2004
PPIB AS	VP(meU)*(fC)*(meA)(fC)(meG)(fA)(meU)(fG)(meG)(fA)(meA)(fU)(meU)(fU)(meG)(fC)(meU)(fG)*(meU)*(fU)	437	20	Reynolds et al. 2004
NTC S	(fC)*(MeA)*(fA) (MeU) (fU) (MeG) (fC) (MeA) (fC) (MeU) (fG) (MeA) (fU) (MeA) (fA) (MeU) (fG)*(MeA)*(fA)	NA	19	Hoogenboezem et al. 2024
NTC AS	VP(MeU)*(fU)*(MeC) (fA) (MeU) (fU) (MeA) (fU) (MeC) (fA) (MeG) (fU) (MeG) (fC) (MeA) (fA) (MeU)*(fU)*(MeG)	NA	19	Hoogenboezem et al. 2024
ASO^{Htt}	CTCGActaaagcaggATTTC	4042	20	Kordasiewicz et al. 2012
Htt PNA probe	5’/N Cy3-OO-TATATCAGTAAAGAGATTAA 3'/C	NA	20	Alterman et al. 2015
NTC PNA probe	5'/N Cy3-OO-GGGACTGGCTAGTTAAACA 3'/C	NA	19	Hoogenboezem et al. 2024
Key: S=sense strand, AS=anti-sense strand, *=phosphorothioate, Me=2’-O-Methyl, f=2’fluoro, VP=vinyl phosphonate, O = ethylene glycol linker, lowercase = DNA, for ASO^{Htt} uppercase = 2’-O-(2-methoxy)ethyl				

Table S1: Nucleic acid sequences used
